# Programmed cell death ligand-1-mediated enhancement of hexokinase 2 expression is inversely related to T-cell effector gene expression in non-small-cell lung cancer

**DOI:** 10.1186/s13046-019-1407-5

**Published:** 2019-11-12

**Authors:** Sehui Kim, Ji-Young Jang, Jaemoon Koh, Dohee Kwon, Young A. Kim, Jin Chul Paeng, Chan-Young Ock, Bhumsuk Keam, Miso Kim, Tae Min Kim, Dae Seog Heo, Doo Hyun Chung, Yoon Kyung Jeon

**Affiliations:** 1 Department of Pathology, Seoul National University College of Medicine, 103 Daehak-ro, Jongno-gu, Seoul, 03080 Republic of Korea; 2 Department of Biomedical Sciences, Seoul National University College of Medicine, Seoul, Republic of Korea; 3 Cancer Research Institute, Seoul National University, Seoul, Republic of Korea; 4 Bioinfra Life Science Inc., Seoul, Republic of Korea; 5 Department of Pathology, Seoul Metropolitan Government-Seoul National University Boramae Medical Center, Seoul, Republic of Korea; 6 Department of Nuclear Medicine, Seoul National University Hospital, Seoul National University College of Medicine, Seoul, South Korea; 7 Department of Internal Medicine, Seoul National University College of Medicine, Seoul, Republic of Korea

**Keywords:** Programmed cell death-ligand-1, Hexokinase 2, Glycolysis, Non-small cell lung cancer, Tumor microenvironment

## Abstract

**Background:**

We investigated the role of PD-L1 in the metabolic reprogramming of non-small cell lung cancer (NSCLC).

**Methods:**

Changes in glycolysis-related molecules and glycolytic activity were evaluated in PD-L1^low^ and PD-L1^high^ NSCLC cells after transfection or knockdown of *PD-L1*, respectively. Jurkat T-cell activation was assessed after co-culture with NSCLC cells. The association between PD-L1 and immune response-related molecules or glycolysis were analyzed in patients with NSCLC and The Cancer Genome Atlas (TCGA).

**Results:**

Transfecting *PD-L1* in PD-L1^low^ cells enhanced hexokinase-2 (HK2) expression, lactate production, and extracellular acidification rates, but minimally altered GLUT1 and PKM2 expression and oxygen consumption rates. By contrast, knocking-down *PD-L1* in PD-L1^high^ cells decreased HK2 expression and glycolysis by suppressing PI3K/Akt and Erk pathways. Interferon-γ (IFNγ) secretion and activation marker expression was decreased in stimulated Jurkat T-cells when co-cultured with HK2-overexpressing vector-transfected tumor cells rather than empty vector-transfected tumor cells. Immunohistochemistry revealed that PD-L1 expression was positively correlated with HK2 expression in NSCLC (*p* < 0.001). In TCGA, *HK2* exhibited a positive linear association with *CD274* (PD-L1) expression (*p* < 0.001) but an inverse correlation with the expression of *CD4*, *CD8A*, and T-cell effector function-related genes in the *CD274*^high^ rather than *CD274*^low^ group. Consistently, there were fewer CD8^+^ T-cells in PD-L1^positive^/HK2^high^ tumors compared to PD-L1^positive^/HK2^low^ tumors in squamous cell carcinoma.

**Conclusions:**

PD-L1 enhances glycolysis in NSCLC by upregulating HK2, which might dampen anti-tumor immunity. PD-L1 may contribute to NSCLC oncogenesis by inducing metabolic reprogramming and immune checkpoint.

## Background

The programmed cell death-1 (PD-1)/ programmed cell death-ligand (PD-L) pathway acts as an immune checkpoint for tumor cells to evade host immune surveillance [1, 2]. Recently, PD-1/PD-L1 blockade has emerged as a therapeutic strategy for patients with cancer [2], and has been approved for the first-line and second-line treatment of patients with non-small-cell lung cancer (NSCLC) [3]. However, some NSCLC patients respond to PD-1/PD-L1 blockade; thus, predictive biomarkers have been identified including PD-L1 expression, tumor mutational burden, and preexisting adaptive immune responses [4]. Although immunohistochemistry for PD-L1 has been approved as a companion/complementary diagnostics for PD-1/PD-L1 blockade, the response rate to PD-1/PD-L1 blockades is approximately 30% even in patients with PD-L1^positive^ NSCLC [5]. Thus, more studies are needed to understand the biology and the mechanism of action of PD-1/PD-L1 blockade.

Most research into the PD-1/PD-L1 pathway has focused on the PD-1/PD-L1 interaction between target cells and immune cells. However, some studies have suggested other functions for PD-1 and PD-L1 beyond immune suppression. Intrinsic PD-L1 signaling promotes tumor cell proliferation and growth in melanoma and ovarian cancer cells by regulating autophagy and the mTOR pathway, and protects tumor cells from interferon (IFN)-mediated cytotoxicity [6–8]. In addition, PD-1 signaling alters T-cell metabolism by inhibiting glycolysis and amino acid metabolism and promoting lipolysis and fatty acid oxidation, thereby inhibiting effector T-cell differentiation [9].

To meet the metabolic requirements for growth and proliferation, tumor cells predominantly use glucose through aerobic glycolysis rather than oxidative phosphorylation (metabolic reprogramming known as the “Warburg effect”) [10]. Metabolic reprogramming is also important for proliferation and effector function of T-cells [11, 12]. Upon activation, lymphocytes undergo a metabolic transition from oxidative phosphorylation to aerobic glycolysis [12–14], which indicates that immune and tumor cells utilize similar metabolic reprogramming for their survival and function. Thus, they could compete for restricted nutrients within the tumor microenvironment (TME) and increased nutrient consumption by tumor cells could lead to an immunosuppressive TME by dampening the T-cell metabolism.

Using a murine sarcoma model, Chang et al. recently reported that PD-L1 elevated glycolysis in tumor cells, which promoted tumor progression by reducing glycolytic capacity and IFN-γ production in T-cells [15]. However, the role of PD-L1 in glucose metabolism and its clinical implication in human cancer cells is unclear. Therefore, we investigated the functional relationship between PD-L1 expression and glycolysis and its regulatory mechanisms in human lung cancer.

## Materials and methods

### Cell culture and reagents

NSCLC cell lines, including A549 and H460, were purchased from the American Type Culture Collection (Manassas, VA, USA). Jurkat cells were purchased from the Korean Cell Line Bank (Seoul, Republic of Korea). Cells were cultured in DMEM (A549 cells) and RPMI 1640 (H460 and Jurkat cells) supplemented with 10% FBS, 100 U/mL penicillin and 100 μg/mL streptomycin in a humidified atmosphere of 5% CO_2_ at 37 °C. 2-deoxy-D-glucose, LY294002, U0126, and SB203580 were purchased from Sigma-Aldrich (St. Louis, MO, USA).

### Patients and samples

Tissue microarrays were constructed from formalin-fixed paraffin-embedded tumor tissues from 393 patients who underwent surgery for NSCLC at Seoul National University Hospital (SNUH, Seoul, Republic of Korea), including 228 pulmonary adenocarcinomas (pADCs) and 165 pulmonary squamous cell carcinomas (pSqCCs). An additional cohort of 80 patients with NSCLCs who received PD-1 blockade immunotherapy was established. Response to PD-1 blockade was assessed based on RECIST v1.1 [16]. Clinicopathological features of patients were analyzed as described in Additional file 1: Supplementary Methods. This study followed the World Medical Association Declaration of Helsinki recommendations and was approved by the Institutional Review Board (IRB) of SNUH (No.: H-1404-100-572).

### Transfection of plasmid vector and siRNAs

A human PD-L1-expressing plasmid (Catalog No. HG10084-UT; Beijing, China) and a human HK2-expressing plasmid Catalog No. HG17967-UT; Beijing, China) were purchased from Sino Biological Inc.. PD-L1-specific siRNA-1 and 2 complementary to PD-L1 (GeneBank accession No.: NM 014143.2) were designed and synthesized by Bioneer (Daejeon, Republic of Korea). The sequences of siRNAs were as follows: PD-L1 siRNA-1, sense 5′-CAGCAUUGGAACUUCUGAU(dTdT)-3′ and antisense 5′-AUCAGAAGUUCCAAUGCUG(dTdT)-3′; PD-L1 siRNA-2, sense 5′-GAAUCAACACAACAACUAA(dTdT)-3′ and antisense 5′-UUAGUUGUUGUGUUGAUUC(dTdT)-3′. For transfection, cells were plated in either 12-well plates or 24-well plates and allowed to adhere for 24 h. Then the cells were transfected with plasmid or siRNA using Lipofectamine 2000 (Invitrogen, Carlsbad, CA, USA) in Opti-MEM media (Qiagen, Germantown, MD, USA). After 6 h, the media were replaced with fresh complement media and cells were harvested 24–48 h after transfection.

### Quantitative reverse transcription PCR (qRT-PCR)

Total RNA was extracted from cells using TRIzol reagent (Life Technologies, Carlsbad, CA, USA) and subjected to reverse transcription using a PrimeScript First Strand cDNA Synthesis Kit (Takara Bio, Otsu, Japan). PCR was performed using SYBR® qRT-PCR Kit (Clontech Laboratories, Mountain View, CA, USA) and a Step One Plus thermal cycler (Applied Biosystems, Foster City, CA, USA) in triplicate. GAPDH and β-actin were used as the internal control. The following primer sequences were used for qRT-PCR: *GAPDH* forward 5′-CCCTTCATTGACCTACCTCAACTACAT-3′ and reverse 5′-ACGATACCAAAGTTGTCATGGAT-3′; *β-actin* forward 5′- CTGGAACGGTGAAGGTGAC-3′ and reverse 5′-AAGGGACTTCCTGTAACAATGCA -3′; *CD274* (PD-L1) forward 5′-TATGGTGGTGCCGACTACAA-3′ and reverse 5′-TGGCTCCCAGAATTACCAAG-3′; *SLC2A1* (GLUT1) forward 5′-GATTGGCTCCTTCTCTGTGG-3′ and reverse 5′-TCAAAGGACTTGCCCAGTTT-3′; *HK2* forward 5′-CAAAGTGACAGTGGGTGTGG-3′ and reverse 5′-GCCAGGTCCTTCACTGTCTC-3′; *PKM2* forward 5′-CCACTTGCAATTATTTGAGGAA-3′ and reverse 5′-GTGAGCAGACCTGCCAGACT-3′; *PFKP* forward 5′-GGGCCAAGGTGTACTTCATC-3′ and reverse 5′-TGGAGACACTCTCCCAGTCG-3′; *PFKL* forward 5′-GGTGGACCTGGAGAAGCTG-3′ and reverse 5′- GGCACCCACATAAATGCC-3′; *PFKM* forward 5′-GCCATCAGCCTTTGACAGA-3′ and reverse 5′-CTCCAAAAGTGCCATCACTG-3′; *GPI* forward 5′- GGAGACCATCACGAATGCAGA − 3′ and reverse 5′-TAGACAGGGCAACAAAGTGCT-3′; *PGK* forward 5′-AAGTCGGTAGTCCTTATGAGC-3′ and reverse 5′- CACATGAAAGCGGAGGTTCT-3′.

### Western blotting

Total cellular proteins were extracted using lysis buffer (5 mM EDTA, 300 mM NaCl, 0.1% NP-40, 0.5 mM NaF, 0.5 mM Na3VO4, 0.5 mM PMSF, and 10 μg/mL each of aprotinin, pepstatin, and leupeptin; Sigma-Aldrich). A total of 30–50 μg protein was separated using 10% SDS-PAGE and transferred to polyvinylidene difluoride membranes (Millipore, Bedford, MA, USA). Then immunoblotting was performed using antibodies against PD-L1 (clone E1L3N), GLUT1, HK2, PKM2, P-Akt, Akt (Cell Signaling Technology, Danvers, MA, USA), P-Erk, Erk, P-p38MAPK, p38MAPK, and β-actin (Santa Cruz Biotechnology, Dallas, TX, USA). Most images of western blots were from parallel gels and actin images were obtained from the stripped and re-probed blots. The immunoblots were visualized using an enhanced chemiluminescence detection system (Amersham Pharmacia Biotech, Uppsala, Sweden).

### Glycolysis assessment: lactate production, hexokinase activity, and extracellular acidification rate (ECAR) assays

Glycolysis was evaluated using lactate production, hexokinase activity, and ECAR assays, as detailed in the Additional file 1: Supplementary Material and Methods.

### Co-culture assay

Direct co-culture and **t**ranswell co-culture system were performed. Co-culture experiments were performed in 24-well plates without or with pore size 0.4 μm insets (Corning Costar, Corning, NY, USA). A549 cell (5 × 10^4^) were seeded and cultured in the outer wells of 24-well plates in DMEM supplemented with 10% FBS for 24 h. A549 cells were transfected with empty or HK2-expressing vectors, as mentioned above. After 24 h, when fully upregulated HK2 expression, medium was changed to RPMI supplemented with 10% FBS and 1% penicillin/streptomycin. Incubating tumor cells for another 24 h, Jurkat cells (4 × 10^5^) were added to directly to tumor cells or added to inner wells in transwell system. After 1 h of stabilization time, final concentration of 2 μg/ml soluble anti-CD3 (eBioscience, San Diego, CA, USA), 1 μg/ml soluble anti-CD28 (eBioscience) and 5 μg/ml anti-mouse Ig (SouthernBiotech, Birmingham, AL, USA) were added. 24 h later, media was harvested for IFN-γ ELISA assay and Jurkat cells were harvested for flow cytometry.

### Enzyme-linked immunosorbent assay (ELISA) for IFN-γ

IFN-γ level in cell-free media was estimated using Human IFN-γ ELISA kit (R&D system, #DY285–05, Minneapolis, MN, USA) according to the manufacturer’s protocol.

### Flow cytometry

Cells were harvested and washed with FACS buffer (0.5% BSA and 0.05% sodium azide in PBS). For discriminating dead cells, cells were first stained with zombie aqua (#423101, Biolegend, San Diego, CA, USA) in PBS for 10 min at room temperature in dark. Cells were stained with the PE-cy7 conjugated anti-CD69 antibody (#310911, Biolegend), PE-conjugated PD-L1 antibody (#329705, Biolegend) in FACS buffer for at least 30 min at 4 °C in dark. Flow cytometry was carried out on the LSRFortessa X-20 (BD Biosciences). Data were analyzed using FlowJo v10.1 (Treestar).

### Immunohistochemistry (IHC)

IHC was performed for PD-L1 (clone E1L3N), HK2, GLUT1, PKM2, and CD8, as detailed in Additional file 1: Supplementary Material and Methods. PD-L1 positivity was defined as ≥10% of tumor cells with moderate-to-strong membranous intensity. H-scores for GLUT1, HK2 and PKM2 were estimated by integrating the intensity and proportion of staining [17]. CD8^+^ TILs per mm^2^ were automatically enumerated, as described in Additional file 1: Supplementary Material and Methods.

### RNA seq and gene set enrichment analyses (GSEAs)

A549 cells transfected with PD-L1-expressing plasmid and H460 cells transfected with PD-L1 siRNA were submitted to RNA seq and GSEAs, as detailed in Additional file 1: Supplementary Material and Methods.

### The Cancer Genome Atlas (TCGA) data analyses

The level 3 data of TCGA were downloaded from the UCSC Cancer Browser (https://genome-cancer.ucsc.edu) including pADC (*N* = 513) and pSqCC (*N* = 502) datasets and genome analysis was performed, as described in Additional file 1: Supplementary Material and Methods.

### Statistical analyses

Statistical analyses were performed using SPSS v.21 (IBM Corp., New York, NY, USA), as detailed in Additional file 1: Supplementary Material and Methods. Two-sided *p* values < 0.05 were considered statistically significant in all analyses.

## Results

### PD-L1 upregulates HK2 expression and aerobic glycolysis in lung cancer cells

GLUT1 is the most common glucose transporter in humans. HK2 and PKM2 catalyze the first and final step of glycolysis, respectively [10, 18, 19]. To investigate whether PD-L1 affects the glycolysis in human NSCLC, we screened basal expression of PD-L1 and glycolysis-related molecules and glycolytic activity in several NSCLC cell lines (Additional file 3: Figure S1A-E). PD-L1^low^ A549 cells showed lower HK2 expression and decreased aerobic glycolysis, as determined using lactate production, hexokinase activity, and ECAR assays, whereas PD-L1^high^ H460 cells showed higher HK2 expression and increased glycolysis (Additional file 3: Figure S1F-J). Thus, we overexpressed PD-L1 in PD-L1^low^ A549 cells and knocked-down PD-L1 in PD-L1^high^ H460 cells for further experiments.

**Fig. 1:**
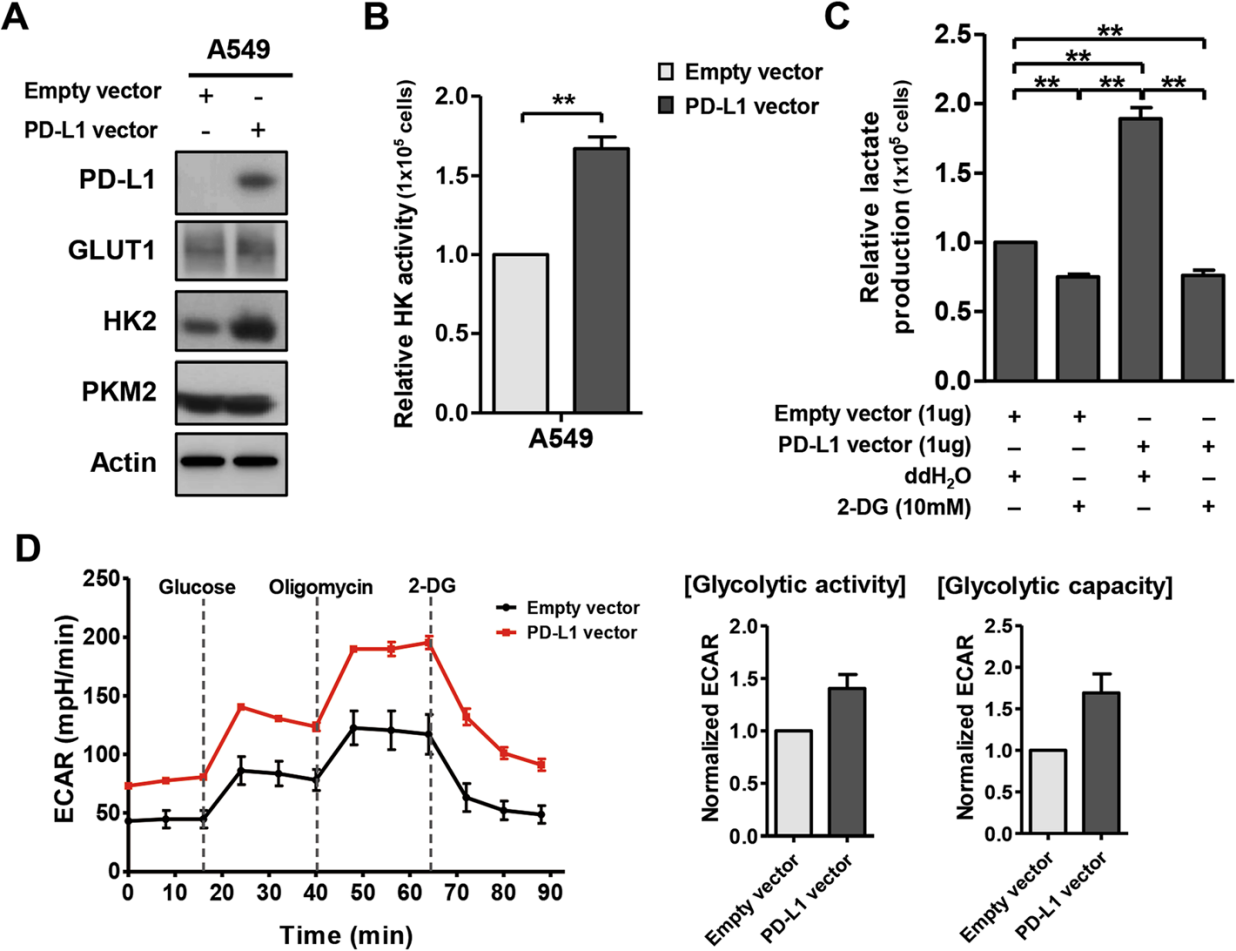
PD-L1 overexpression increases the expression and activity of HK2 and glycolysis in lung cancer cells. PD-L1^low^ A549 cells were transfected with empty or PD-L1-expressing plasmid. Twenty-four hours after transfection, cells were submitted to Western blotting using antibodies against PD-L1, GLUT1, HK2, PKM2, and β-actin (**a**) or hexokinase activity assay (**b**). Lactate production was measured in the absence (ddH_2_O) or presence of 2-deoxy-glucose (2-DG) in A549 cells after PD-L1 overexpression (**c**). ECAR assays were performed in A549 cells transfected with empty or PD-L1-expressing plasmid upon adding glucose, oligomycin, and 2-DG to the culture media (**d**). Histograms represent values normalized to control. Data represent the means ± SEMs of at least three independent experiments or are representative of three independent experiments. All *p* values were calculated using unpaired Student’s *t*-tests and one-way ANOVA. **p < 0.001

**Fig. 2:**
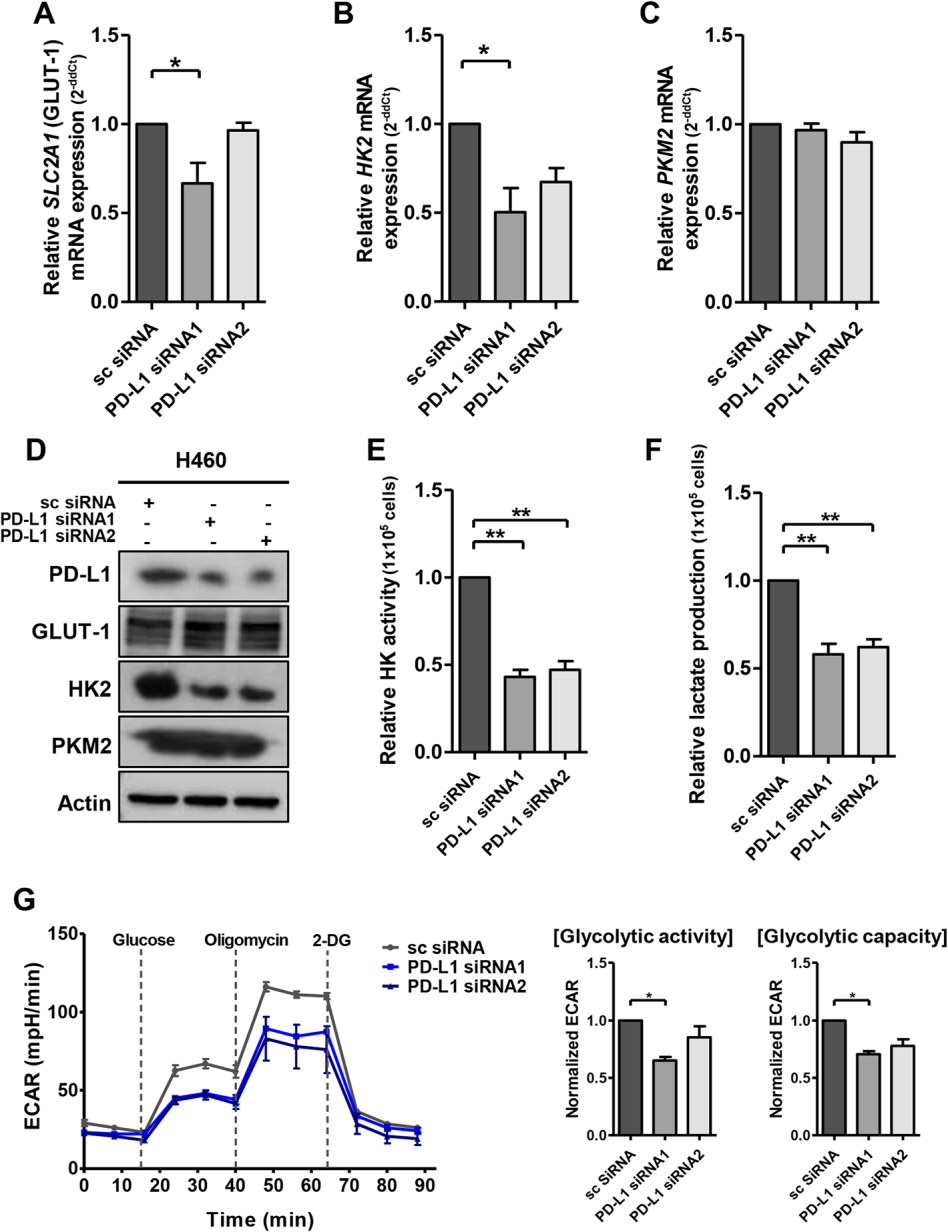
PD-L1 knockdown decreases the expression and activity of HK2 and glycolysis in lung cancer cells. H460 cells were transfected with scrambled control siRNA (sc), PD-L1-specific siRNA1, or 2. Forty-eight hours after transfection, mRNA and protein levels of GLUT1, HK2, and PKM2 were evaluated using qRT-PCR and Western blotting (**a-d**). Hexokinase activity assays (**e**), lactate production assays (**f**), and ECAR assays (**g**) were also performed. Histograms represent values normalized to control. Data represent the means ± SEMs of at least three independent experiments or are representative of three independent experiments. All *p* values were calculated using unpaired Student’s *t*-tests and one-way ANOVA. *p < 0.05; **p < 0.001

Upon PD-L1 overexpression in A549 cells, HK2 expression and hexokinase activity were increased (Fig. 1a, b). Moreover, PD-L1 overexpression significantly increased lactate production and ECAR, which was suppressed by 2-deoxy-D-glucose, a glucose analog that inhibits hexokinase (Fig. 1c, d). By contrast, downregulation of PD-L1 in H460 cells decreased HK2 expression, hexokinase activity, lactate production, and ECAR (Fig. 2). Oxygen consumption rate (Additional file 3: Figure S2) and transcription of other glycolytic genes were minimally affected by PD-L1 expression (Fig. 2a-c; Additional file 3: Figure S3). Consistently, gene set enrichment analyses revealed that the glycolytic pathway was enhanced in A549 cells after PD-L1 transfection and downregulated in H460 cells after PD-L1 knockdown (Additional file 3: Figure S4).

**Fig. 3:**
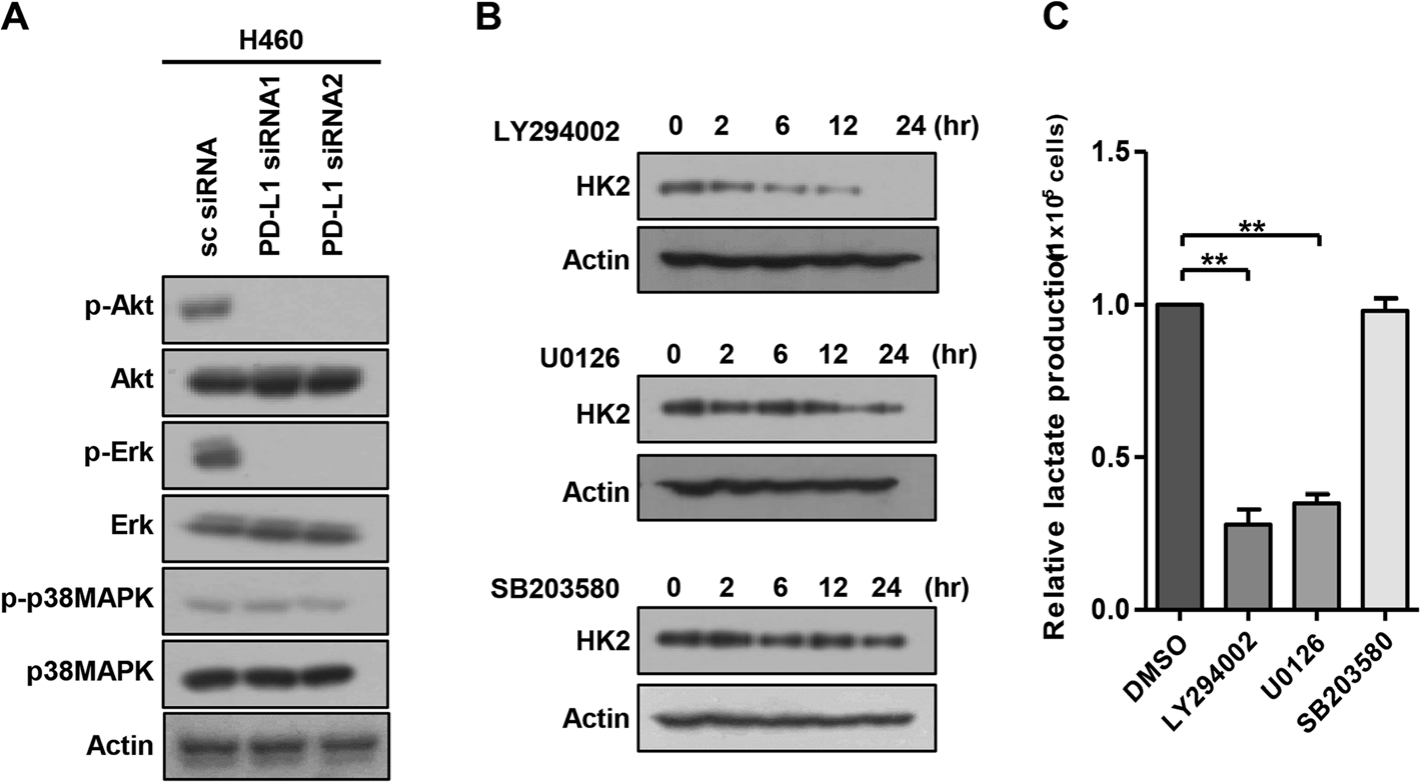
PD-L1 upregulates the expression of HK2 via the PI3K/Akt and Erk signaling pathways. **a** H460 cells were transfected with scrambled control siRNA (sc), PD-L1-specific siRNA1, or 2. Forty-eight hours after transfection, cells were analyzed by Western blotting using antibodies against P-Akt, Akt, P-Erk, Erk, P-p38MAPK, p38MAPK and β-actin. **b** H460 cells were treated with LY294002 (a PI3K inhibitor, 10 μM), U0126 (an Erk inhibitor, 10 μM), or SB203580 (a p38MAPK inhibitor, 10 μM) and analyzed by Western blotting for HK2 and β-actin. **c** H460 cells were treated with LY294002, U0126, or SB203580 and submitted to lactate production assays. Histograms represent the values normalized to control. Data represent the means ± SEMs of at least three independent experiments or are representative of three independent experiments. All *p* values were calculated using one-way ANOVA. **p < 0.001

To explore signaling pathways involved in PD-L1-mediated HK2 up-regulation, we evaluated several pathways that control glucose metabolism. PD-L1 knockdown decreased Akt and Erk phosphorylation but minimally altered p38MAPK phosphorylation in H460 cells (Fig. 3a). Consistently, HK2 expression and lactate production were decreased by a PI3K/Akt inhibitor (LY294002) and an Erk inhibitor (U0126) but not by a p38MAPK inhibitor (SB203580) (Fig. 3b, c). Together, these results suggest that PD-L1 upregulates HK2 expression via the PI3K/Akt and Erk pathways, thereby increasing glycolytic activity in human lung cancer cells.

### PD-L1 expression is positively correlated with HK2 expression in NSCLCs from patients

**Fig. 4:**
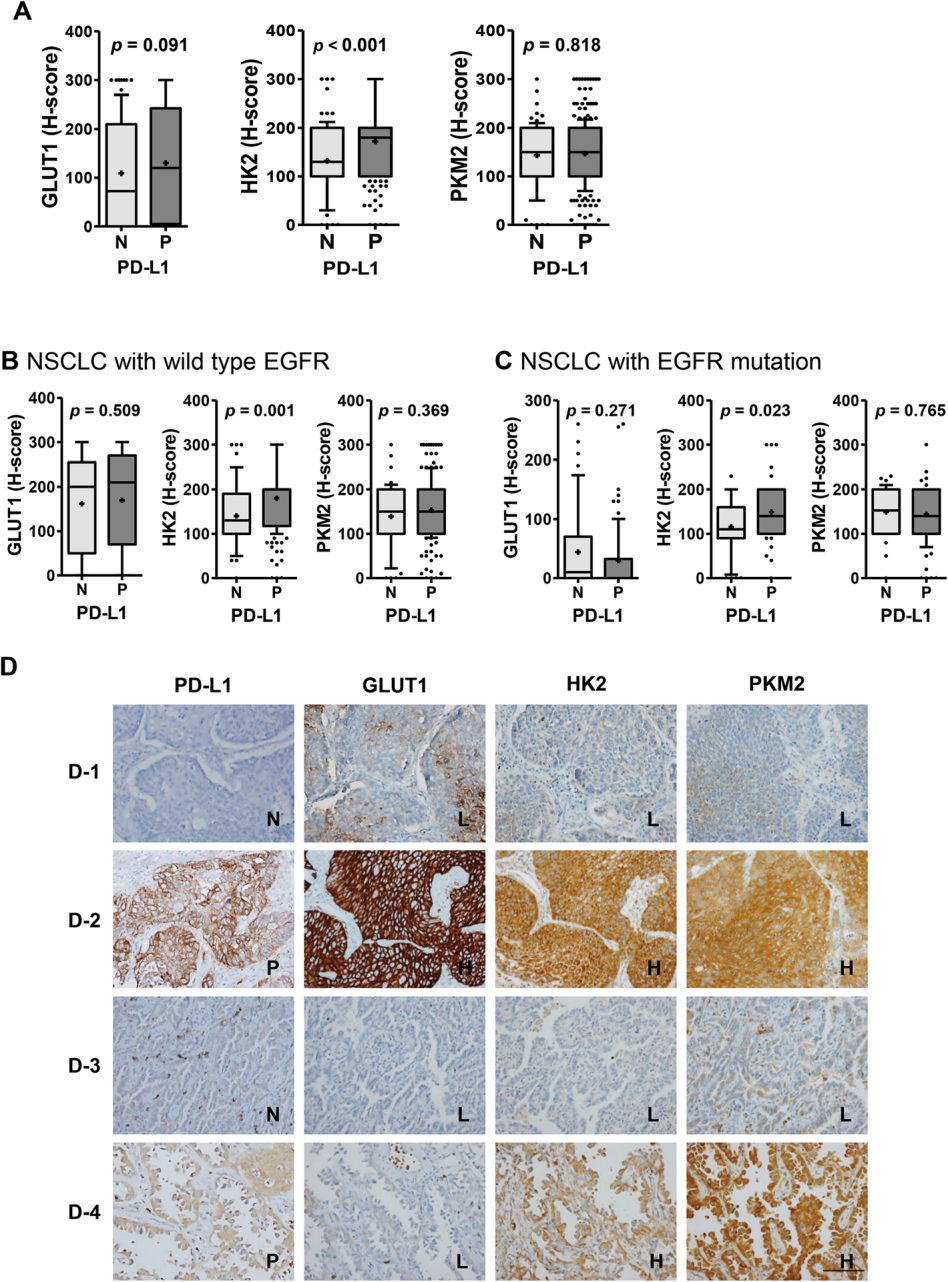
Positive correlation between the expression of PD-L1 and HK2 in human lung cancer tissues. Immunohistochemistry (IHC) analyses for GLUT1, HK2, and PKM2 were performed using tumor tissues from NSCLC patients (*N* = 393). **a** The expression (H-score) of these molecules was compared between PD-L1^negative^ and PD-L1^positive^ NSCLCs and statistical differences were analyzed using Mann Whitney U-tests. The expression of these molecules was also compared between PD-L1^negative^ and PD-L1^positive^ cases in patients with **b** wild-type *EGFR* (*N* = 230) and **c** mutated *EGFR* (*N* = 126). Statistically differences were analyzed using Mann Whitney U-tests. The whiskers are drawn from the 10th percentile to the 90th percentile. The midline of the box is the median and “+” denotes the mean. Points below and above the whiskers are individual points. **d** Representative IHC images are as follows: D-1, a pSqCC case that is PD-L1^negative^ and expresses low levels of GLUT1, HK2, and PKM2; D-2, a pSqCC case that is PD-L1^positive^ and expresses high levels of GLUT1, HK2, and PKM2; D-3, a pADC case that is PD-L1^negative^ and expresses low levels of GLUT1, HK2, and PKM2; D-4, a pADC case that is PD-L1^positive^ and expresses low level of GLUT1 and high levels of HK2 and PKM2. Cases were separated into low and high expression group using the median H-score of each molecule as a cutoff. Cases with PD-L1 score 2 and 3 (moderate to strong membranous staining in ≥10% of tumor cells) was designated as PD-L1^positive^. (original magnification × 400, bar = 100 μm). Abbreviations: N, negative; P, positive; L, low; H, high

Next, the correlation between PD-L1 expression and glycolysis in human NSCLCs was investigated by immunohistochemistry and FDG-PET in 393 patients with NSCLC (Additional file 2: Table S1). Consistent with previous reports [20], all PET indices and expression of GLUT1 and HK2 were higher in pSqCC than pADC (all *p* < 0.001) (Additional file 3: Figure S5). Of note, only HK2 expression was significantly higher in PD-L1^positive^ NSCLC compared to PD-L1^negative^ NSCLC (*p* < 0.001) (Fig. 4). This positive correlation between the expression of PD-L1 and HK2 was observed in NSCLCs with wild-type *EGFR* and in those with mutated *EGFR* (*p* = 0.001 and *p* = 0.023, respectively) (Fig. 4b, c), and in both pSqCC and pADC (*p* < 0.001 and *p* = 0.009, respectively) (Additional file 3: Figure S6). In TCGA analyses, *HK2* mRNA levels also exhibited a significant positive correlation with *CD274* mRNA levels in NSCLC (Spearman *rho* = 0.189, *p* < 0.001; data not shown).

### Increased tumoral HK2 expression impairs T-cell effector function

**Fig. 5:**
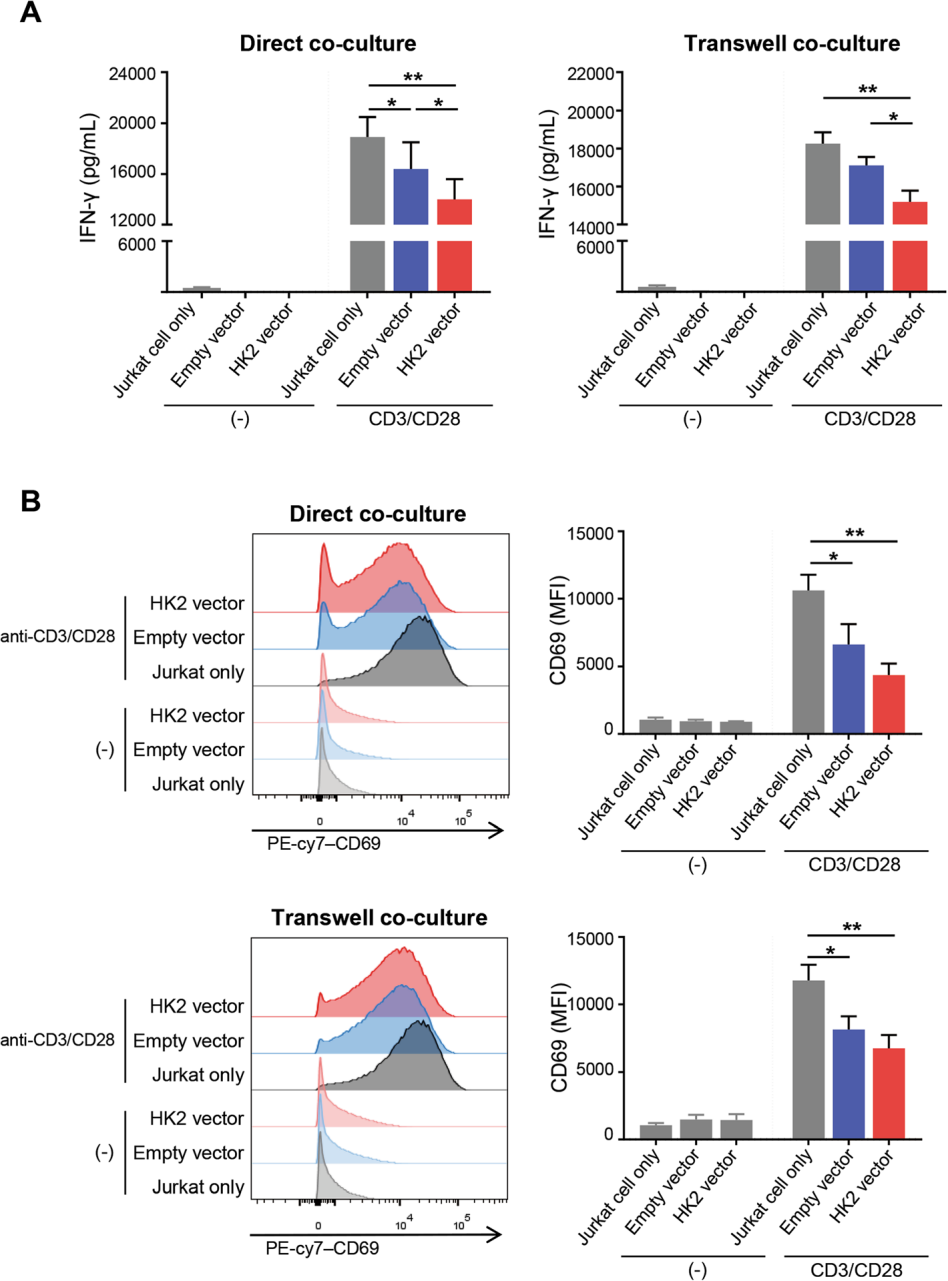
Upregulated HK2 expression in tumor cells impairs effector T-cell function. Direct co-culture and transwell co-culture system were adopted to investigate effector T-cell function impairment. Jurkat cells were cultured only (“Jurkat only”) or co-cultured with either empty vector transfected A549 cells (“empty vector”) or HK2 vector transfected A549 cells (“HK2 vector”). IFN-γ secretion was measured using ELISA (**a**) and CD69 expression using flow cytometry (**b**) in the absence or the presence of anti-CD3/CD28 stimulation. Data represent the means ± SEMs of at least three independent experiments or are representative of three independent experiments. Statistical significance was calculated using Two-way ANOVA with Tukey multiple comparisons test. *p < 0.05; **p < 0.001

To explore the biological and clinical relevance of the PD-L1-mediated upregulation of HK2 and glycolysis in NSCLC, Jurkat cells were co-cultured with A549 cells. As previously demonstrated, PD-L1 upregulates tumoral glycolysis/HK2 expression and therefore, we tried to find the outcomes of upregulated tumoral HK2 expression in anti-tumor immunity. We transfected HK2 low A549 cell lines with an empty vector and a HK2 vector and then, co-cultured with Jurkat cells using direct and transwell co-culture systems. After 24 h CD3/CD28 activation, we analyzed the IFN-γ secretion and activation marker expression on Jurkat cells for assessing the effector T-cell function (Fig. 5). In both of direct and transwell co-culture systems, IFN-γ secretion and CD69 expression, an activation marker were highest in Jurkat cells without tumor cell co-culture (“Jurkat only”). When co-culture with tumor cells, IFN-γ secretion was lower in Jurkat cells co-cultured with HK2 overexpressing-A549 cells (“HK2 vector”) than in Jurkat cells co-cultured with empty vector transfected-A549 cells (“empty vector”) in both direct and transwell co-culture systems (*p* < 0.05; Fig. 5a). In addition, CD69 showed similar tendency in both systems (Fig. 5b). These results suggested that tumoral HK2 upregulation, probably subsequent glycolysis upregulation, might impair effector T-cell functions in tumor microenvironment.

### HK2 expression and glycolytic signature are inversely related to the expression of T-cell effector molecules in PD-L1^high^ human NSCLCs

**Fig. 6:**
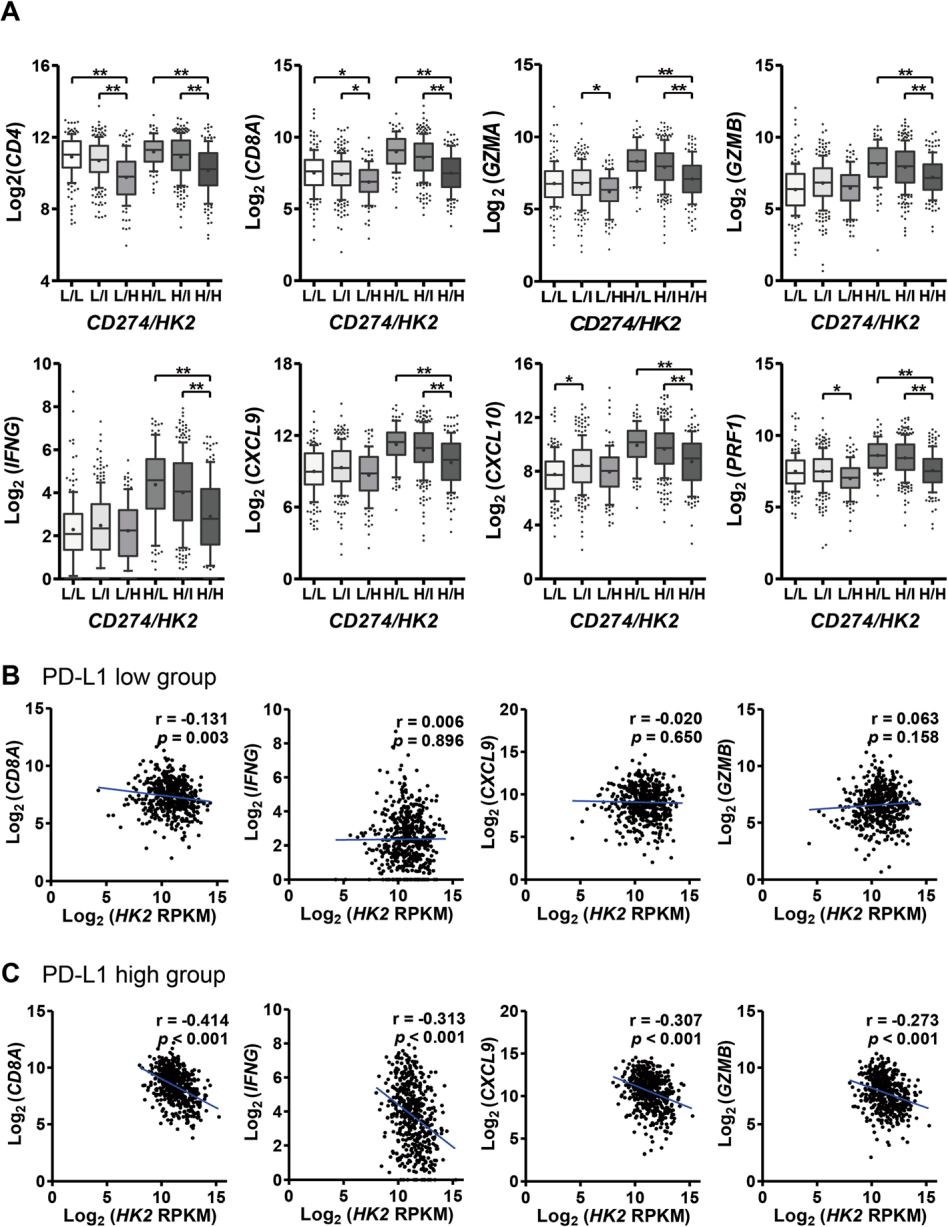
HK2 expression is inversely correlated with the expression of T-cell effector molecules in human lung cancers, particularly in those with high PD-L1 expression. **a** The expression levels of T-cell effector molecules, including *CD4, CD8A, GZMA, GZMB, IFNG, CXCL9, CXCL10* and *PRF1*, were assessed according to *CD274* (PD-L1) and *HK2* expression status in NSCLCs from TCGA dataset (*N* = 1015). Cases were dichotomized into PD-L1^low^ and PD-L1^high^ groups based on median values and then trichotomized into HK2^low^ (<25th percentile), HK2^intermediate^ (25-75th percentile), and HK2^high^ (>75th percentile) groups. Statistical differences were analyzed using Kruskal-Wallis tests. **b** and **c** The expression levels of T-cell effector molecules were compared according to *HK2* expression status in NSCLCs with low *CD274* (PD-L1) mRNA levels (**b**) and with high *CD274* (PD-L1) mRNA levels (**c**) in NSCLCs from TCGA dataset. Statistical significance was calculated using Pearson’s correlation analyses. *p < 0.05; **p < 0.001. Abbreviations: L, low; I, intermediate; H, high

To validate above findings, we analyzed the relationships between the expression of *HK2* and T-cell effector response-related genes using TCGA data. *HK2* expression was inversely correlated with immune-related genes (*CD4*, Pearson *r* = − 0.304, *p* < 0.001; *CD8A*, *r* = − 0.170, *p* < 0.001; *GZMA*, *r* = − 0.116, *p* < 0.001; *PRF1*, *r* = − 0.130, *p* < 0.001) (data not shown). Next, we compared the expression of immune-related genes according to the combined expression status of *CD274* (PD-L1) and *HK2*. Notably, the expression of all immune-related genes exhibited a significant inverse correlation with *HK2* in the PD-L1^high^ group rather than the PD-L1^low^ group (Fig. 6). A trend toward an inverse correlation between the expression of *SLC2A1* (GLUT1) or *PKM* and immune-related genes was also observed in PD-L1^high^ NSCLCs, but less consistently than that observed with *HK2* (Additional file 3: Figure S7).

**Fig. 7:**
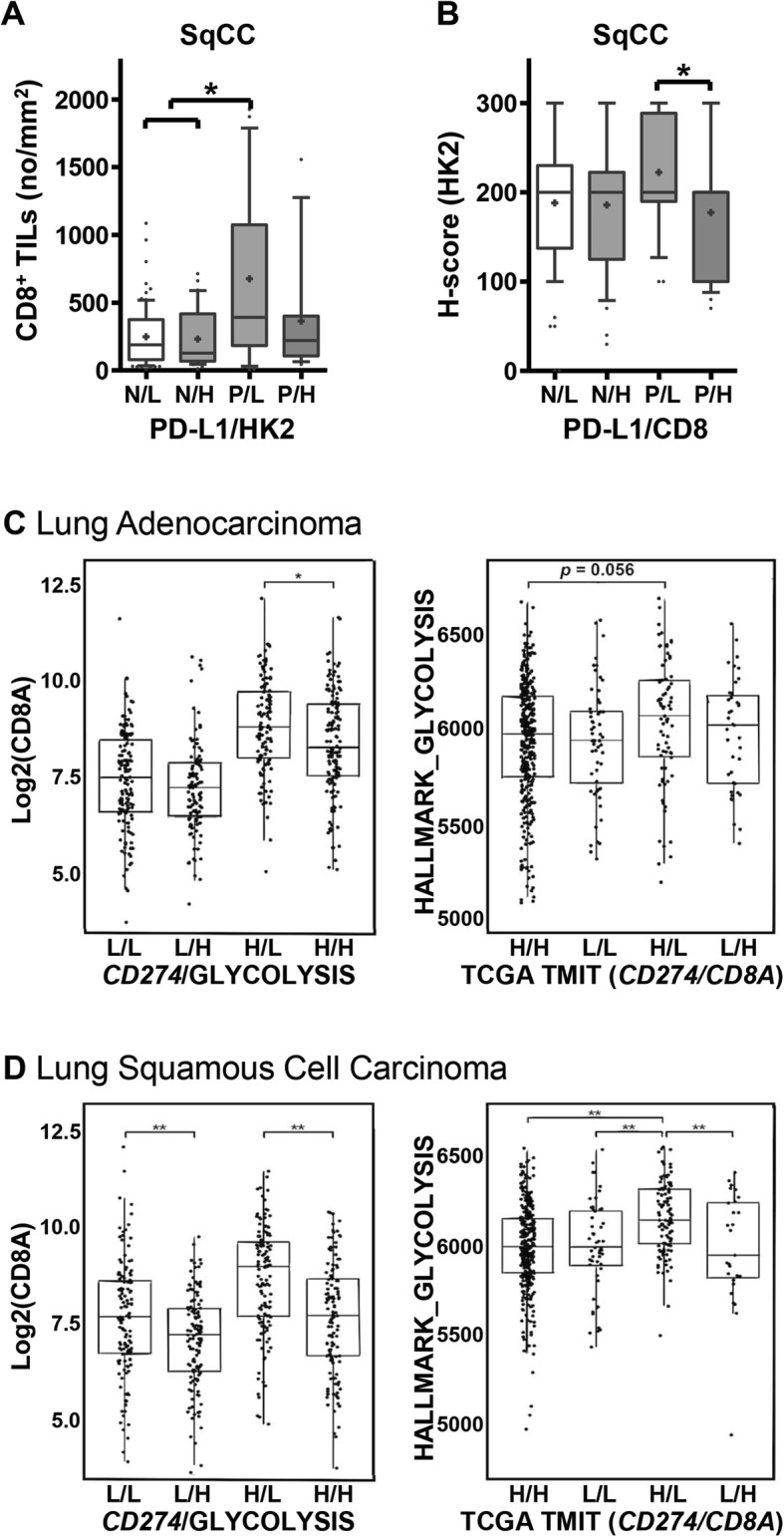
High HK2 expression is inversely correlated with the number of CD8^+^ TILs in patients samples and high glycolysis signature is inversely related with *CD8A* expression in TCGA dataset. **a** The numbers of CD8^+^ TILs were evaluated according to PD-L1 and HK2 expression status using tumor tissues from patients with pSqCC. Statistical significance was calculated using Kruskal-Wallis tests. **b** HK2 expression was evaluated according to PD-L1 expression and the numbers of CD8^+^ TILs using tumor tissues from patients with pSqCC. Statistical significance was calculated using Kruskal-Wallis tests. **c** & **d** *CD8A* transcript levels were compared according to *CD274* (PD-L1)/glycolysis signature and the glycolysis signature was compared according to the tumor microenvironment immune type (TMIT) based on *CD274* (PD-L1)*/CD8A* expression status **c** in pADC and **d** in pSqCC from TCGA dataset. Statistical differences were analyzed using Kruskal-Wallis tests. *p < 0.05; **p < 0.001. Abbreviations: N, negative; P, positive; L, low; H, high

In our patient cohort, high numbers of CD8^+^ TILs were observed in patients with PD-L1^high^ NSCLC (*p* < 0.05, data not shown), whereas the number of CD8^+^ TILs tended to be inversely correlated with HK2 expression in PD-L1^positive^ NSCLCs. Furthermore, the number of CD8^+^ TILs was highest in PD-L1^positive^/HK2^low^ pSqCCs (Fig. 7a), and HK2 expression was significantly higher in PD-L1^positive^/CD8^+^ TIL^low^ pSqCCs compared to PD-L1^positive^/CD8^+^ TIL^high^ pSqCCs (Fig. 7b). In TCGA data, *CD8A* expression was also higher in CD274^high^/glycolytic-signature^low^ NSCLCs compared to CD274^high^/glycolytic-signature^high^ NSCLCs (Fig. 7c, d). The glycolytic signature and *HK2* expression were highest in NSCLCs of so-called tumor microenvironment immune type (TMIT) III (Fig. 7c, d and Additional file 3: Figure S8), which express high levels of PD-L1 but low levels of CD8, thus being considered poor responders to anti-PD-1/PD-L1 immunotherapy [21]. Together, these data indicate that HK2 expression was inversely correlated to the expression of T-cell effector molecules and the number of CD8^+^ TILs in PD-L1^positive^ NSCLCs. This suggests that increased HK2 expression might negatively affect the T-cell effector function in PD-L1^positive^ NSCLC.

### High HK2 expression in NSCLC tended to be associated with poor response to anti-PD-1 immunotherapy in patients

Finally, to evaluate the effects of HK2 overexpression on the response to PD-1/PD-L1-targeted immunotherapy, we analyzed 80 NSCLC patients who were treated with nivolumab (*N* = 63) or pembrolizumab (*N* = 17). The characteristics of the patients are detailed in Additional file 2: Table S2. The median duration of follow-up was 5.2 months (1.0–49.9 months) and the overall response rate (ORR) was 16.3% (13/80). Tumors that exhibited the lowest and highest quartile of HK2 expression were designated HK2^low^ and HK2^high^ cases, respectively. The ORR was higher in the PD-L1^positive^ group (26.5% [9/34]) than the PD-L1^negative^ group (8.7% [4/46]). In addition, the ORR was higher in the PD-L1^positive^/HK2^low^ group than the PD-L1^positive^/HK2^high^ group, although statistically insignificant (Additional file 3: Figure S9). There was no significant difference in patient survival according to PD-L1 and HK2 status (data not shown). These findings suggest that patients with HK2^high^ NSCLC might show poor response to anti-PD-1 immunotherapy despite PD-L1 expression. However, further validation is required.

## Discussion

Several studies have found that HIF-1α enhances glycolysis by upregulating glycolytic enzymes [22, 23], and upregulates PD-L1 [24], which suggests a potential link between PD-L1 and glycolysis in cancer. The current study demonstrates that PD-L1 upregulates aerobic glycolysis in NSCLC cells by enhancing HK2 expression. This is the first study to demonstrate the intrinsic effects of PD-L1 on HK2-mediated glycolysis in human lung cancer cells. Moreover, we found a significant positive correlation between PD-L1 and HK2 expression in human NSCLC tissues. Although oncogenic mutations affect tumor cell metabolism [10, 25], the relationship of PD-L1 and HK2 expression was not affected by *EGFR* mutation status in NSCLCs. However, it remains unclear whether mutations in other genes affect link between PD-L1 and HK2 in NSCLCs. Meanwhile, TCGA analyses showed a positive correlation between PD-L1 and HK2 expression in hepatocellular carcinoma (*rho* = 0.461, *p* < 0.001), sarcoma (*rho* = 0.188, *p* < 0.001), and stomach adenocarcinoma (*rho* = 0.225, *p* < 0.001), but not in renal cell carcinoma and urothelial carcinoma (data not shown). These findings suggest that a functional link between PD-L1 and HK2 might occur in various cancers in a histologic type-dependent manner.

In this study, we confirmed that the changes in surface PD-L1 as well as total PD-L1 expression after PD-L1 overexpression and knockdown (Figs. 1 and 2 and Additional file 3: Figure S2A). Therefore, there is a potential that surface PD-L1 expression might give an intrinsic signal to modulate metabolism of tumor cells. By treating murine tumor cells with anti-PD-L1 blocking antibodies, Chang et al. previously showed that surface PD-L1 might be involved in tumor cell glycolysis [15]. In this study, we demonstrated that upregulation of total PD-L1 expression raised glycolysis/HK2 expression of lung cancer cells in the absence of PD-1 ligation. However, we did not compare the actual effect of surface and intracellular PD-L1 expressions on HK2 expression and glycolysis of human lung cancer, and thus it is unclear if both surface and cytoplasm PD-L1 or only surface PD-L1 might be involved in the PD-L1-mediated increase in HK2 overexpression and glycolysis in tumor cells. This is an intriguing issue and remains to be determined in further studies incorporating biochemical assays.

Of note, we also found that upregulated tumoral HK2 expression impaired an effector T-cell function (i.e. IFN-γ production), in a co-culture system. These findings were further validated using TCGA data and IHC for lung cancer tissues. *HK2* expression was inversely correlated with the expression of T-cell effector response-related genes and the number of CD8^+^ TILs in PD-L1^high^ NSCLCs but not PD-L1^low^ NSCLCs. These findings suggest that the expression status of PD-L1 and HK2 in tumor cells might be involved in the regulation of immune response in the TME of NSCLCs. However, since the correlations between variables were often weak to moderate despite statistical significance in analysis of TCGA and patients tissue data, the cause-effect relationship between variables needs to be interpreted with caution. Despite of this limitation, our results regarding the relationship between PD-L1, HK2, and T-cell effector function suggests that combination analyses of HK2 and PD-L1 expression in tumor cells might provide useful information to predict the TME immune signature in NSCLC.

When classifying TME immune types (TMIT) based on PD-L1 expression and CD8^+^ T-cell, tumors can be divided into four groups: PD-L1^high^/CD8^+^ T-cell^high^ (TMIT I), PD-L1^low^/CD8^+^ T-cell^low^ (TMIT II), PD-L1^high^/CD8^+^ T-cell^low^ (TMIT III), and PD-L1^low^/CD8^+^ T-cell^high^ (TMIT IV). Patients with TMIT I tumors are considered the best candidates for PD-1/PD-L1-targeted therapy [21, 26]. By contrast, those with TMIT III tumors exhibit a poor response to PD-1/PD-L1 blockade despite high PD-L1 expression [21]. The current study demonstrates that PD-L1^high^/CD8^+^ T-cell^low^ TMIT III NSCLCs could be characterized by high HK2 expression and glycolytic signature.

Regarding functional aspects of the immune responses, our results suggest that PD-L1-mediated HK2/glycolysis upregulation of tumor cells might dampen T-cell function in the TME of NSCLCs. In previous studies, tumor-derived lactate, a metabolite of glycolysis, inhibits T-cell function [27], and upregulates PD-L1 expression via the lactate receptor-TAZ pathway in lung cancer, thereby contributing to T-cell suppression [28]. Taken together, these and the current findings suggest a potential positive feedback loop of the glycolysis/lactate-PD-L1-HK2-glycolysis axis in the TME. Thus, it is feasible that tumor cells might survive and outcompete surrounding TILs in the TME by expressing PD-L1 through metabolic reprogramming for survival/proliferation, dampening TIL functions by rendering a metabolically harmful microenvironment, and giving a direct inhibitory signal to PD-1 on adjacent immune cells, as schematically summarized in Additional file 3: Figure S10. Thus, the metabolic status of tumor cells could affect the responsiveness of patients to anti-PD-1/PD-L1 immunotherapy. To address this issue, we evaluated the effects of HK2 overexpression in tumor cells on the clinical response to PD-1 blockades. We observed a trend toward a better response in patients with PD-L1^positive^/HK2^low^ NSCLC compared to those with PD-L1^positive^/HK2^high^ NSCLCs, but no difference in the survival time between these groups. These results might be attributable to small number of patients, variable performance status and previous treatment history, laboratory-developed PD-L1 test, limitations of retrospective evaluation, and a possible occurrence of resistance mechanisms other than HK2 overexpression. Therefore, the clinical value of HK2 as a potential metabolic biomarker for anti-PD-1/PD-L1 therapy needs further validation.

Moreover, HK2 itself could be a therapeutic target in cancer therapy. HK2 participates in aerobic glycolysis and can affect many other metabolic processes through its product, glucose-6-phosphate. Besides metabolic process, HK2 has been demonstrated to prohibit apoptosis. HK2 binds to a voltage-dependent anion channel (VDAC) and the HK2/VDAC interaction stabilizes mitochondrial membrane and prevents binding pro-apoptotic factors to VDAC [29–31]. Therefore, HK2 inhibitors or in vivo deletion by CRISPR/Cas9 [32] could affect tumor cells by inhibiting tumor metabolism and promoting apoptosis. Applying the concept of combinational therapy for cancer [33, 34], HK2 inhibition could enhance the response rate of PD-1/PD-L1 therapy by releasing immune cells from metabolic competition and directly increasing tumor cell apoptosis. This hypothesis needs to be validated by future studies.

## Conclusions

In summary, this study demonstrates that PD-L1 increases glycolysis in NSCLC cells by upregulating HK2, which is associated with reduced expression of T-cell effector genes. These findings suggest that PD-L1 expression might enhance tumor cell survival by increasing glycolysis intrinsically and providing inhibitory signals to neighboring T-cells extrinsically, through interaction with PD-1 and metabolic competition. Thus, HK2 expression and glycolysis could be potential biomarkers or therapeutic targets in NSCLC patients in the era of cancer immunotherapy.

## Supplementary information

Additional file 1: Supplementary material and methods. More detailed information about Patients and samples, FDG-PET/CT image analysis, Glycolysis assays, Oxygen consumption rate (OCR) assay, RNAseq and Gene set enrichment analyses, TCGA analyses, Immunohistochemistry (IHC) and Statistical analysis was addressed. (DOCX 45 kb)

Additional file 2: **Table S1.** Clinicopathological parameters of patients with NSCLCs. **Table S2** Clinicopathological features of NSCLC patients with PD-1 blockade. (DOCX 21 kb)

Additional file 3: **Figure S1.** The expressions of HK2 and glycolytic activity are elevated in PD-L1^high^ lung cancer cell lines. **Figure S2.** PD-L1 overexpression or knockdown does not affect oxidative phosphorylation. **Figure S3.** PD-L1 overexpression or knockdown does not affect the mRNA level of glycolysis-related genes, other than *HK2*. **Figure S4.** PD-L1 expression is positively correlated with glycolysis signature in NSCLC cells. **Figure S5.** Glycolysis-related parameters analyzed by PET scanning and immunohistochemistry in NSCLC patients. **Figure S6.** Basal expression of glycolysis-related molecules according to PD-L1 expression in pSqCC and pADC patients. **Figure S7.** Expression levels of T-effector immune response-related genes according to *CD274* (PD-L1*)* and *SLC2A1* (GLUT1*)* or *PKM* expression status in NSCLC from TCGA data. **Figure S8.** HK2 mRNA is higher in TIMT III (CD274^high^/HK2^low^) than in TIMT I (CD274^high^/HK2^high^) NSCLC from TCGA data. **Figure S9.** High HK2 expression is related to a lower response rate to PD-1 blockade in patients with NSCLC. **Figure S10.** A model figure of this study. (PDF 1278 kb)

## Data Availability

The datasets analyzed during the current study are available from the corresponding author on reasonable request.

## Abbreviations

ECAR: Extracellular acidification rate
IFN: Interferon
IHC: Immunohistochemistry
NSCLC: Non-small cell lung cancer
OCR: Oxygen consumption rate
pADC: Pulmonary adenocarcinoma
PD-1: Programmed cell death protein 1
PD-L1: Programmed death-ligand 1
pSqCC: Pulmonary squamous cell carcinoma
sc: Scramble
siRNA: Small interfering RNA
TCGA: The Cancer Genome Atlas
TME: Tumor microenvironment
TMIT: Tumor microenvironment immune type
