# Supplementary material for: Programmed cell death ligand-1-mediated enhancement of hexokinase 2 expression is inversely related to T-cell effector gene expression in non-small-cell lung cancer
Source: J Exp Clin Cancer Res. 2019 Nov 12;38:462. doi: 10.1186/s13046-019-1407-5 (PMC6852926; doi:10.1186/s13046-019-1407-5)
Supplement: Supplementary file 1 — Supplementary material and methods. More detailed information about Patients and samples, FDG-PET/CT image analysis, Glycolysis assays, Oxygen consumption rate (OCR) assay, RNAseq and Gene set enrichment analyses, TCGA analyses, Immunohistochemistry (IHC) and Statistical analysis was addressed. (DOCX 45 kb) [file 13046_2019_1407_MOESM1_ESM.docx]

**Supplementary Material and Methods**

**Patients and samples**

Pathologic diagnosis was performed according to the current 4^th^ World Health Organization (WHO) classification of lung tumors.^1^ Pathologic tumor-node-metastasis (TNM) staging was based on the 7^th^ American Joint Committee on Cancer.^2^ *EGFR* and *KRAS* mutations were examined using direct sequencing or peptide nucleic acid-clamping PCR, while *ALK* translocation was evaluated using fluorescence *in situ* hybridization (FISH).^3^ FDG-PET/CT images were acquired and SUVmax and TLG values were obtained as previously described,^4^ as detailed in below paragraph.

**FDG-PET/CT image analysis**

In all patients, FDG-PET/CT images were acquired according to our standard imaging protocol using dedicated PET/CT scanners (Biograph 40 or Biograph 64, Siemens Healthcare, Germany). Details of the standard imaging protocol were described previously.^4^ PET/CT images were reviewed by two nuclear medicine physicians. In FDG-PET, a standardized uptake value (SUV) is generally considered a quantitative parameter of metabolism, and SUVmax describes the maximal SUV of a tumor lesion. The total lesion glycolysis (TLG) of a lesion was calculated as mean SUV (SUVmean) × metabolic tumor volume (MTV), which reflects both the metabolic activity and tumor burden. The MTV for each threshold margin was measured by setting the margin thresholds as 40% and 2.5 of SUVmax for each lesion. In the image analyses, a spheroidal volume of interest (VOI) was drawn automatically by a dedicated software package (Syngo. via, Siemens Healthcare) with a reproducibility of 100%.

**Glycolysis assays: Lactate production, hexokinase activity, and ECAR assays**

For lactate production assays, cell culture media were collected and diluted 1:100 in lactate assay buffer. Then the amount of lactate present in the medium was estimated using a lactate assay kit (BioVision Research Products, Mountain View, CA, USA) according to the manufacturer’s instructions, by subtracting the amount of lactate in the medium without cells from that in the medium from each sample.

Hexokinase activity assays were performed using a colorimetric hexokinase assay kit (Abcam, Cambridge, UK) according to the manufacturer’s instructions. In cells, glucose is converted into glucose-6-phosphate by hexokinase; then the glucose-6-phosphate is oxidized by glucose-6-phosphate dehydrogenase to form NADH, which reduces a colorless probe to a colored product with strong absorbance at 450 nm. Therefore, the hexokinase activity was estimated by measuring OD 450 nm.

Extracellular acidification rate (ECAR) assays were performed using a Seahorse Extracellular Flux Analyzer XF24e (Seahorse Bioscience Inc., Santa Clara, CA, USA) according to the manufacturer’s instructions. Briefly, cells were seeded in a Seahorse plate and cultured overnight to 80% confluence. The culture media were replaced with cellular assay media supplemented with 1 mM glutamine and incubated for 1 h in a CO_2_-free incubator before measurement. Assays were performed according to Seahorse protocols with final concentrations of 10 mM glucose, 2 μM oligomycin, and 50 mM 2-deoxy-D-glucose (2-DG). All glycolytic assays were performed in at least three independent experiments for each cell line on separate days.

**Oxygen consumption rate (OCR) assay**

OCR assays were performed using a Seahorse Extracellular Flux Analyzer XF24e (Seahorse Bioscience Inc.) according to the manufacturer’s instructions. Briefly, cells were seeded in a Seahorse plate and cultured overnight to 70% confluence. The culture media were replaced with cellular assay media supplemented with 1 mM pyruvate, 2 mM glutamine, and 10 mM glucose, and the plates were incubated for 1 h in a CO2-free incubator before measurement. Assays were performed according to Seahorse protocols with final concentrations of 2 μM oligomycin, 1 μM carbonyl cyanide-4 (trifluoromethoxy) phenylhydrazone (FCCP), and 1 μM antimycin A. OCR assays were performed in three independent experiments for each cell line on separate days.

**RNAseq and gene set enrichment analyses (GSEAs)**

Total RNA was extracted using RNeasy with QIAshredders (Qiagen, Germantown, MD, USA) and the RNA quality was assessed an Agilent RNA 6000 Nano kit (Agilent Technologies, Santa Clara, CA, USA). Ahead of cDNA library construction, 1 ug total RNA and magnetic beads with Oligo (dT) were used to enrich poly (A) mRNA. Then the purified mRNAs were disrupted into short fragments and double-stranded cDNAs were immediately synthesized. The cDNAs were subjected to end-repair poly (A) addition and connected with sequencing adapters using the TruSeq RNA sample preparation kit (Illumina, San Diego, CA, USA). The final library sizes and qualities were evaluated electrophoretically with an Agilent High Sensitivity DNA kit (Agilent Technologies) and the fragments were between 350–450 base pairs. Subsequently, the library was sequenced using an Illumina HiSeq2500 sequencer (Illumina). Gene expression levels were measured with Cufflinks v2.1.1 using the gene annotation database of Ensembl release 77 and differentially expressed genes were identified using the Cuffdiff tool with a default parameter setting with a significance of *p* < 0.05.

GSEA is a computational method used to identify classes of genes that are overexpressed in a large set of genes and compared between PD-L1 low and PD-L1 high cell lines (A549 with PD-L1 overexpression vs. control; H460 with PD-L1 knockdown vs. control). These analyses were performed using GenePattern (https://genepattern.broadinstitute.org/). The Molecular Signatures Database H collection, consisting of canonical pathways and experimental signatures curated from publications, was used for the analyses.

**The Cancer Genome Atlas (TCGA) data analyses**

Genomic analyses were performed to explore the associations among PD-L1, glycolysis-associated molecule, and immune response-related molecules using the level 3 data of TCGA which were downloaded from the UCSC Cancer Browser (https://genome-cancer.ucsc.edu) on June 3, 2015. TCGA data included clinical information and mRNA expression data obtained by RNAseq (Illumina HiSeq V2 platform). First, the lung adenocarcinoma (N = 513) and lung squamous cell carcinoma (N = 502) datasets including mRNA expression were analyzed. To compare the findings from lung cancer with those from other cancer types, the mRNA expression data of other cancer types were also analyzed. Altogether, samples of 32 cancer types (N = 10,327) were included in the analyses.

Glycolysis signature (level) was calculated using single sample GSEA. Tumor microenvironment immune type (TMIT) was assessed previously.^5^ In lung adenocarcinoma and lung squamous cell carcinoma, the correlations between *CD8A* and *CD274* (PD-L1) expression and glycolysis signature or TMIT were analyzed.

**Immunohistochemistry (IHC)**

IHC was performed using TMA and the following antibodies: rabbit anti-PD-L1 (E1L3N) XP^®^ monoclonal (Cell Signaling Technology), rabbit anti-GLUT1 polyclonal *(*Thermo Fisher Scientific, Rockford, IL, USA), rabbit anti-HK2 polyclonal (Genetex, Irvine, CA, USA), rabbit anti-PKM2 polyclonal (Cell Signaling Technology), and rabbit anti-CD8 monoclonal (clone SP16, Thermo Fisher Scientific) antibodies. Immunostaining was performed using the Benchmark XT autostainer (Ventana Medical Systems, Tucson, AZ, USA). The PD-L1 immunohistochemistry were evaluated based on the intensity and proportion of membranous staining in tumor cells and scored as follows: 0, negative; 1, weak or moderate in <10% of tumor cells; 2, moderate in ≥10% of tumor cells; 3, strong (more intense than alveolar macrophages for PD-L1) in ≥10% of tumor cells. Cases with scores of 2 or 3 were designated as PD-L1^positive^. H-scores of GLUT1, HK2 and PKM2 were estimated by integrating the intensity and proportion of staining, as reported previously.^6^ The numbers of CD8^+^ TILs in tumor nests and stroma were estimated automatically by applying modified nuclear IHC algorithms in Aperio ImageScope software (Aperio Technologies, Vista, CA, USA).

**Statistical analyses**

All statistical analyses were performed using SPSS software (version 21; IBM Corp., New York, NY, USA) and images were created using the GraphPad Prism 5 software. For patient sample data and *in vitro* studies, the mRNA and protein expression levels of several continuous variables, including GLUT1 (*SLC2A1*), HK2 (*HK2*), PKM2 (*PKM*), and PET indices were compared using Student *t*-tests, one-way ANOVA, Mann Whitney U-tests, and Kruskal-Wallis tests. For TCGA data, the statistical significance of continuous variables, such as the mRNA levels of *CD274* (PD-L1), *SLC2A1* (GLUT1), *HK2* (HK2), and *PKM,* was calculated using Spearman’s correlation. The mRNA levels of T-cell effector response-related genes according to *CD274*, *SLC2A1*, *HK2,* and *PKM* status were compared using Pearson’s correlation analyses and Kruskal-Wallis tests. Differential response rates to PD-1 immunotherapy were analyzed using Fisher’s exact tests.  Experiments with two variable factors (Interferon-γ releasing assay after co-culture) were subjected to two-way ANOVA, followed by a Tukey’s multiple comparisons test. Two-sided *p* values < 0.05 were considered statistically significant.

**Supplementary References**

1. Travis WD, Brambilla E, Burke AP, Marx A, Nicholson AG. *WHO classification of tumours of the lung, pleura, thymus and heart*. IARC: Lyon, 2015.

2. Compton CC. AJCC cancer staging atlas : a companion to the seventh editions of the AJCC cancer staging manual and handbook. 2012

3. Won JK, Keam B, Koh J, Cho HJ, Jeon YK, Kim TM *et al.* Concomitant ALK translocation and EGFR mutation in lung cancer: a comparison of direct sequencing and sensitive assays and the impact on responsiveness to tyrosine kinase inhibitor. *Ann Oncol* 2015; **26**: 348-354.

4. Keam B, Lee SJ, Kim TM, Paeng JC, Lee SH, Kim DW *et al.* Total Lesion Glycolysis in Positron Emission Tomography Can Predict Gefitinib Outcomes in Non-Small-Cell Lung Cancer with Activating EGFR Mutation. *J Thorac Oncol* 2015; **10**: 1189-1194.

5. Ock CY, Keam B, Kim S, Lee JS, Kim M, Kim TM *et al.* Pan-Cancer Immunogenomic Perspective on the Tumor Microenvironment Based on PD-L1 and CD8 T-Cell Infiltration. *Clin Cancer Res* 2016; **22**: 2261-2270.

6. Koh J, Go H, Keam B, Kim MY, Nam SJ, Kim TM *et al.* Clinicopathologic analysis of programmed cell death-1 and programmed cell death-ligand 1 and 2 expressions in pulmonary adenocarcinoma: comparison with histology and driver oncogenic alteration status. *Mod Pathol* 2015; **28**: 1154-1166.
