# Supplementary material for: Programmed cell death ligand-1-mediated enhancement of hexokinase 2 expression is inversely related to T-cell effector gene expression in non-small-cell lung cancer
Source: J Exp Clin Cancer Res. 2019 Nov 12;38:462. doi: 10.1186/s13046-019-1407-5 (PMC6852926; doi:10.1186/s13046-019-1407-5)
Supplement: Supplementary file 2 — Table S1. Clinicopathological parameters of patients with NSCLCs. Table S2 Clinicopathological features of NSCLC patients with PD-1 blockade. (DOCX 21 kb) [file 13046_2019_1407_MOESM2_ESM.docx]

**Table S1.** Clinicopathological parameters of NSCLC patients.

| Clinicopathological parameter | Adenocarcinoma  (N = 228) (%) | Squamous cell carcinoma  (N = 165) (%) |
| --- | --- | --- |
| Median age at surgery (range) | 63 (36-86) | 66 (43-87) |
| Sex |  |  |
| Male | 104 (45.6) | 158 (95.8) |
| Female | 124 (54.4) | 7 (4.2) |
| Smoking* |  |  |
| Never | 130 (58.0) | 12 (7.3) |
| Ever | 94 (42.0) | 153 (92.7) |
| T stage |  |  |
| T1 | 104 (45.6) | 28 (17.0) |
| T2 | 107 (46.9) | 95 (57.6) |
| T3 | 13 (5.7) | 32 (19.4) |
| T4 | 4 (1.8) | 10 (6.1) |
| N stage* |  |  |
| N0 | 165 (73.3) | 90 (55.2) |
| N1-2 | 60 (36.7) | 73 (44.8) |
| Stage* |  |  |
| I | 139 (61.5) | 55 (34.0) |
| II | 39 (17.3) | 60 (37.0) |
| III | 38 (16.8) | 42 (25.9) |
| IV | 10 (4.4) | 5 (3.1) |
| *EGFR* mutation* |  |  |
| Wild-type | 98 (44.5) | 132 (97.1) |
| Mutated | 122 (55.5) | 4 (2.9) |
| *KRAS* mutation* |  |  |
| Wild-type | 13 (81.3) | 2 (66.7) |
| Mutated | 3 (18.7) | 1 (33.3) |
| *ALK* translocation* |  |  |
| Not translocated | 124 (96.9) | 59 (98.3) |
| Translocated | 4 (3.1) | 1 (1.7) |
| Neoadjuvant treatment* |  |  |
| No | 222 (98.2) | 160 (97.0) |
| Yes | 4 (1.8) | 5 (3.0) |
| Adjuvant treatment* |  |  |
| No | 160 (70.8) | 107 (64.8) |
| CTx and/or RTx | 66 (29.2) | 58 (35.2) |
| PD-L1 IHC score |  |  |
| 0 | 63 (27.6%) | 29 (17.6%) |
| 1 | 44 (19.3%) | 85 (51.5%) |
| 2 | 97 (42.5%) | 38 (23.0%) |
| 3 | 24 (10.5%) | 13 (7.9%) |

*Some cases have missing values.

Abbreviations: ALK, anaplastic lymphoma kinase; CTx, chemotherapy; EGFR, epidermal growth factor receptor; IHC, immunohistochemistry; NSCLC, non-small cell lung cancer; RTx, radiotherapy

**Table S2.** Clinicopathological features of NSCLC patients with PD-1 blockade

| Clinicopathological parameter | N (Total = 80) (%) |
| --- | --- |
| Median age at surgery (range) | 61.5 (32-93) |
| Sex |  |
| Male | 65 (81.2) |
| Female | 15 (18.8) |
| Smoking |  |
| Never | 30 (37.5) |
| Ever | 50 (62.5) |
| Histology |  |
| Adenocarcinoma | 49 (61.3%) |
| Squamous cell carcinoma | 16 (20.0%) |
| Others | 15 (18.8 %) |
| Stage* |  |
| III | 8 (10.1) |
| IV | 71 (89.9) |
| *EGFR* mutation* |  |
| Wild type | 55 (85.9) |
| Mutated | 9 (14.1) |
| *KRAS* mutation* |  |
| Wild type | 26 (86.7) |
| Mutated | 4 (13.3) |
| *ALK* translocation* |  |
| Not translocated | 56 (93.3) |
| Translocated | 4 (6.7) |
| Immunotherapy |  |
| Pembrolizumab | 17 (21.3) |
| Nivolumab | 63 (78.7) |
| Acquisition time of tumor sample |  |
| Naïve therapy | 40 (50.0) |
| On or after chemotherapy | 15 (18.8) |
| Just before immunotherapy | 25 (31.2) |

*Some cases have missing values.

Abbreviations: ALK, anaplastic lymphoma kinase; CTx, chemotherapy; EGFR, epidermal growth factor receptor; NSCLC, non-small cell lung cancer.
