# Supplementary material for: Programmed cell death ligand-1-mediated enhancement of hexokinase 2 expression is inversely related to T-cell effector gene expression in non-small-cell lung cancer
Source: J Exp Clin Cancer Res. 2019 Nov 12;38:462. doi: 10.1186/s13046-019-1407-5 (PMC6852926; doi:10.1186/s13046-019-1407-5)
Supplement: Supplementary file 3 — Figure S1. The expressions of HK2 and glycolytic activity are elevated in PD-L1high lung cancer cell lines. Figure S2. PD-L1 overexpression or knockdown does not affect oxidative phosphorylation. Figure S3. PD-L1 overexpression or knockdown does not affect the mRNA level of glycolysis-related genes, other than HK2. Figure S4. PD-L1 expression is positively correlated with glycolysis signature in NSCLC cells. Figure S5. Glycolysis-related parameters analyzed by PET scanning and immunohistochemistry in NSCLC patients. Figure S6. Basal expression of glycolysis-related molecules according to PD-L1 expression in pSqCC and pADC patients. Figure S7. Expression levels of T-effector immune response-related genes according to CD274 (PD-L1) and SLC2A1 (GLUT1) or PKM expression status in NSCLC from TCGA data. Figure S8. HK2 mRNA is higher in TIMT III (CD274high/HK2low) than in TIMT I (CD274high/HK2high) NSCLC from TCGA data. Figure S9. High HK2 expression is related to a lower response rate to PD-1 blockade in patients with NSCLC. Figure S10. A model figure of this study. (PDF 1278 kb) [file 13046_2019_1407_MOESM3_ESM.pdf]

Supplementary Figures and legends

Supplementary Figure S1

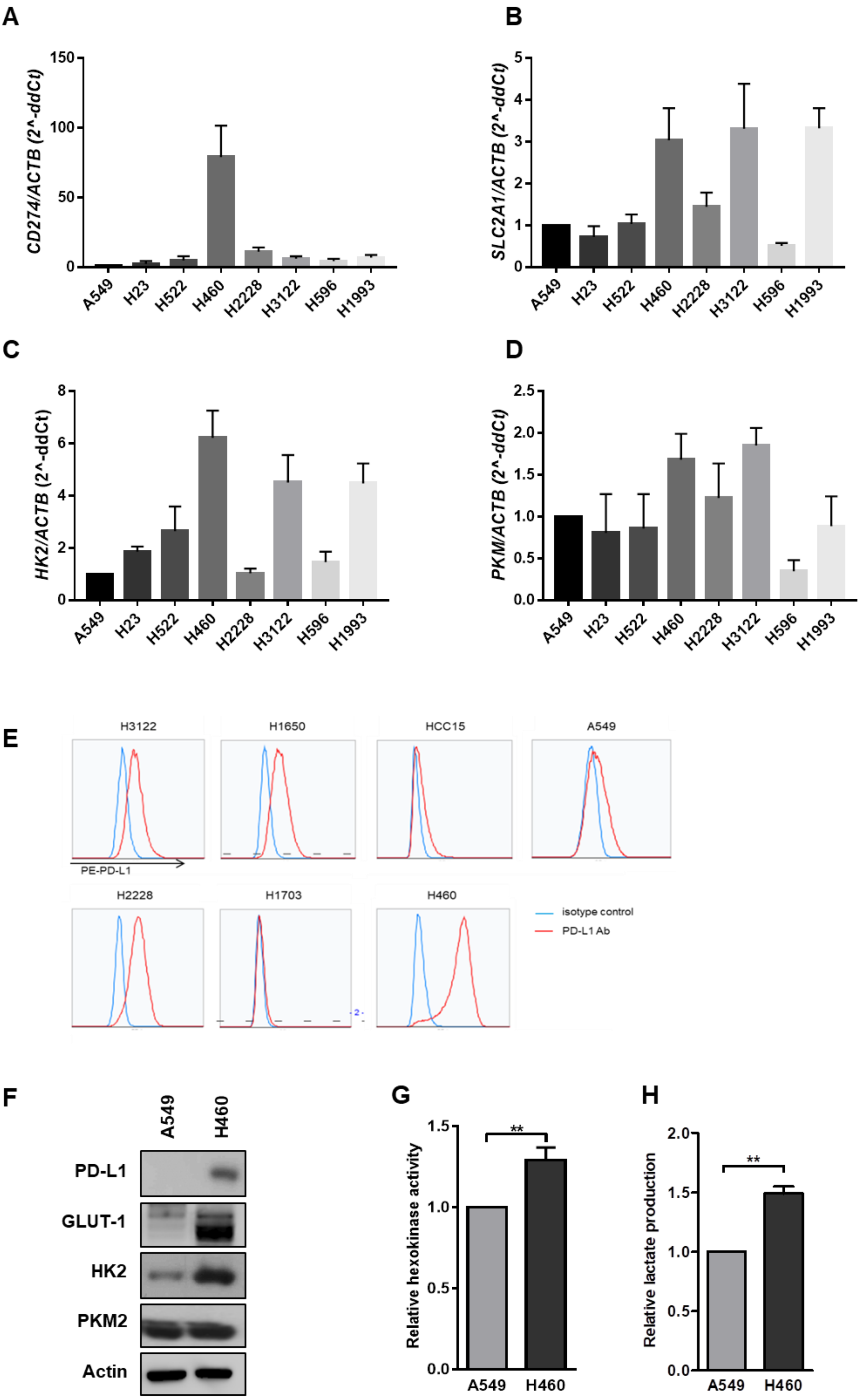

Supplementary Figure S1 *(continued)*

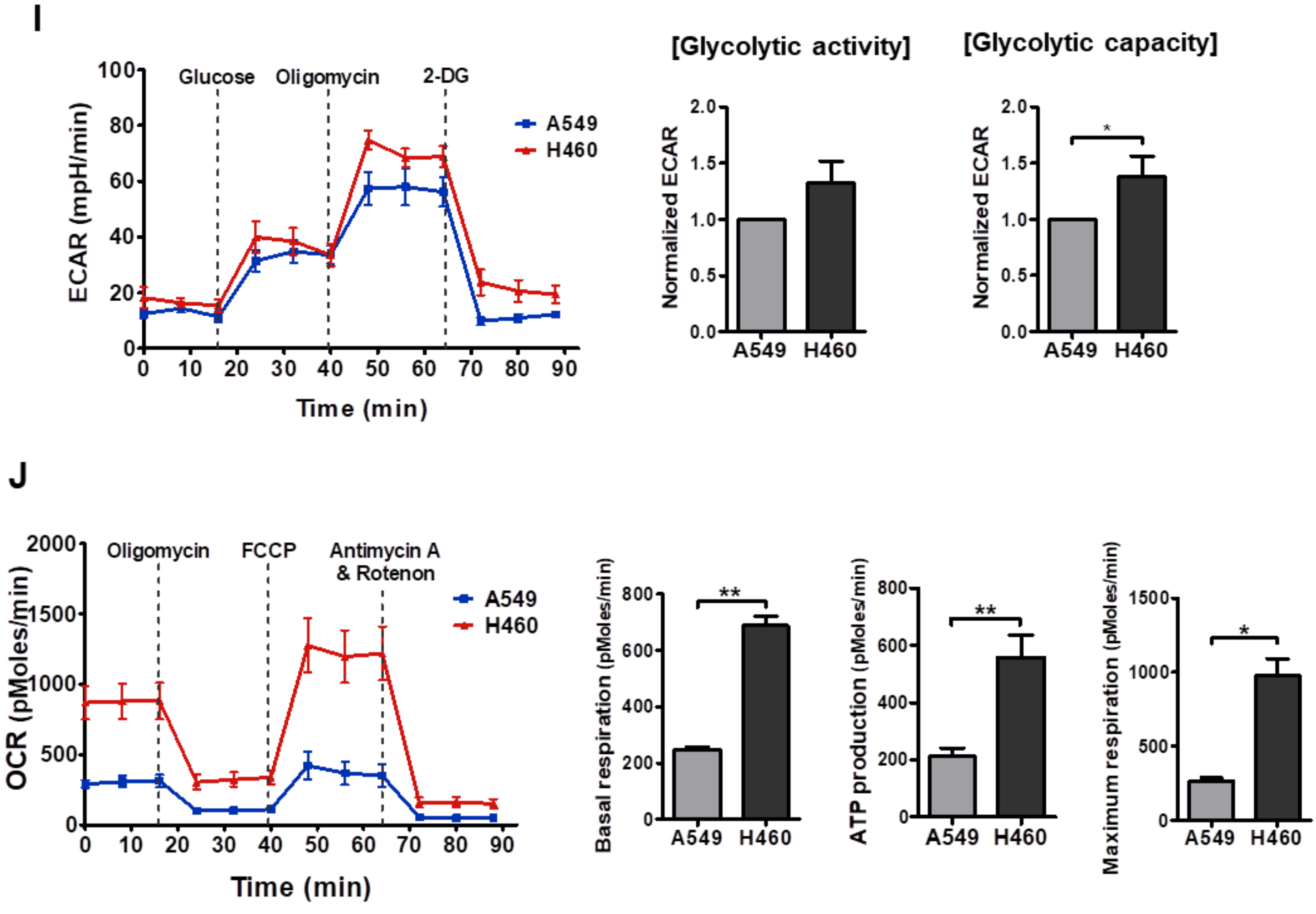

Supplementary Figure S1. The expressions of hexokinase-2 and glycolytic activity are elevated in PD-L1^{high} lung cancer cell lines. The baseline expressions of PD-L1, GLUT1 (*SLC2A1*), hexokinase-2 (*HK2*) and PKM2 (*PKM*) were evaluated using qRT-PCR in eight lung adenocarcinoam cell lines **(A-D)**. The baseline expression of surface PD-L1 expression was evaluated using Flow cytometry **(E)**. The baseline expressions of PD-L1, GLUT1 (*SLC2A1*), hexokinase-2 (*HK2*) and PKM2 (*PKM*) were evaluated using Western blotting **(F)** in A549 and H460 cells. (**G-I)** Hexokinase activity (G), lactate production (H), and extracellular acidification rate (ECAR) (I) were analyzed in A549 and H460 cells. **(J)** Baseline oxidative phosphorylation varies among cell lines. The oxygen consumption rate (OCR) was analyzed and basal respiration, ATP production and maximum respiration, were measured in A549 and H460 cells. Data represent the means ± SEMs of at least three independent experiments or are representative of three independent experiments. Histograms (A-D, G-J) represent the values normalized to control. Data represent the means ± SDs of at least three independent experiments or are representative of three independent experiments. All *p* values were calculated using one-way ANOVA and unpaired Student's *t*-tests. *$p < 0.05$; **$p < 0.001$.

Supplementary Figure S2

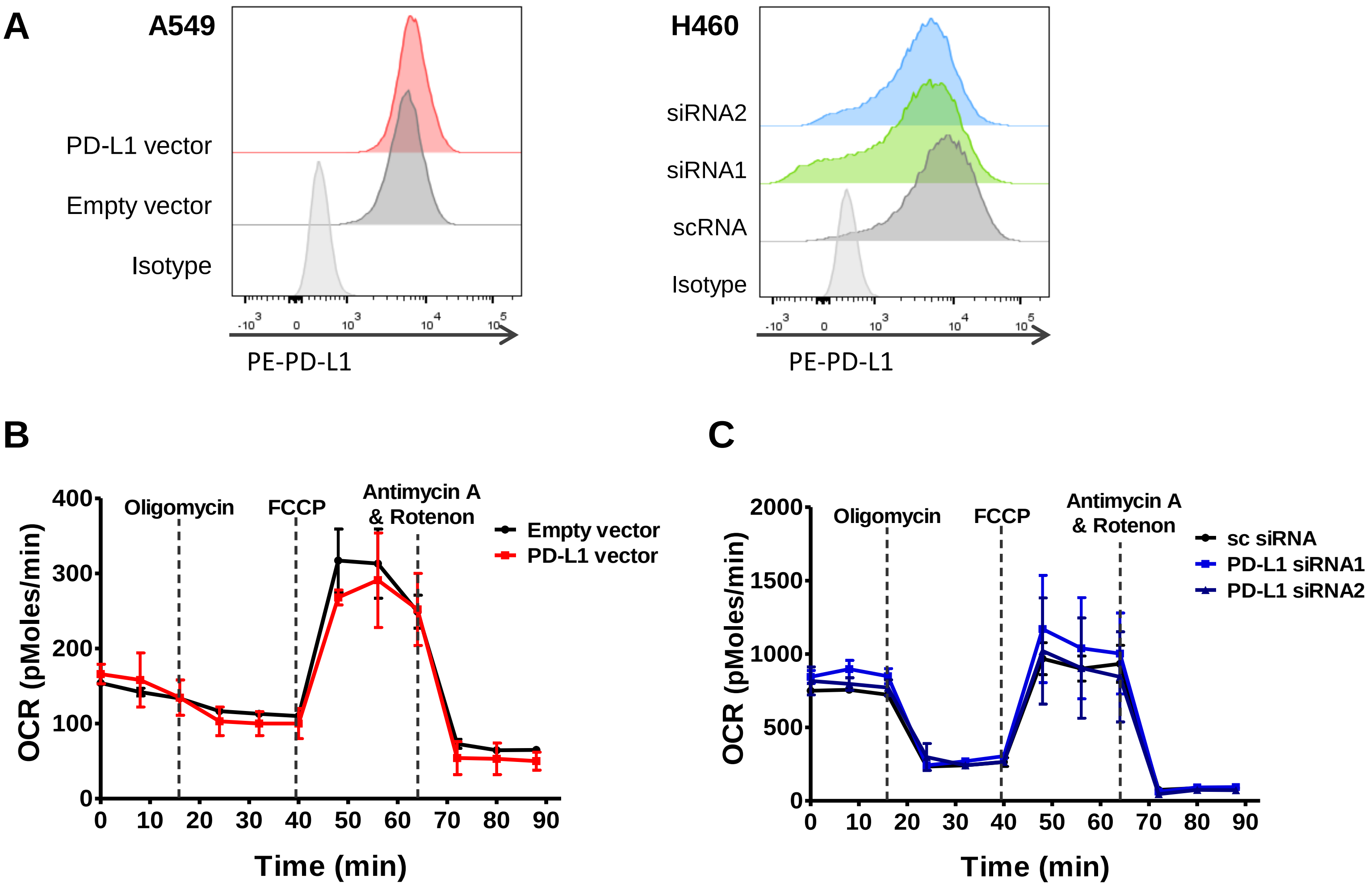

Supplementary Figure S2. PD-L1 overexpression or knockdown does not affect oxidative phosphorylation. (A) Surface PD-L1 expression was upregulated or downregulated by transfecting PD-L1 vector/PD-L1 siRNAs. (B) PD-L1^{low} A549 cells were transfected with empty or PD-L1-expressing vector. Twenty-four hours after transfection, cells were analyzed using OCR assays. **(C)** H460 cells were transfected with scrambled control (sc) siRNA, PD-L1 siRNA1, or PD-L1 siRNA2. Forty-eight hours after transfection, cells were analyzed using OCR assays. Data represent the means ± SEMs of at least three independent experiments.

Supplementary Figure S3

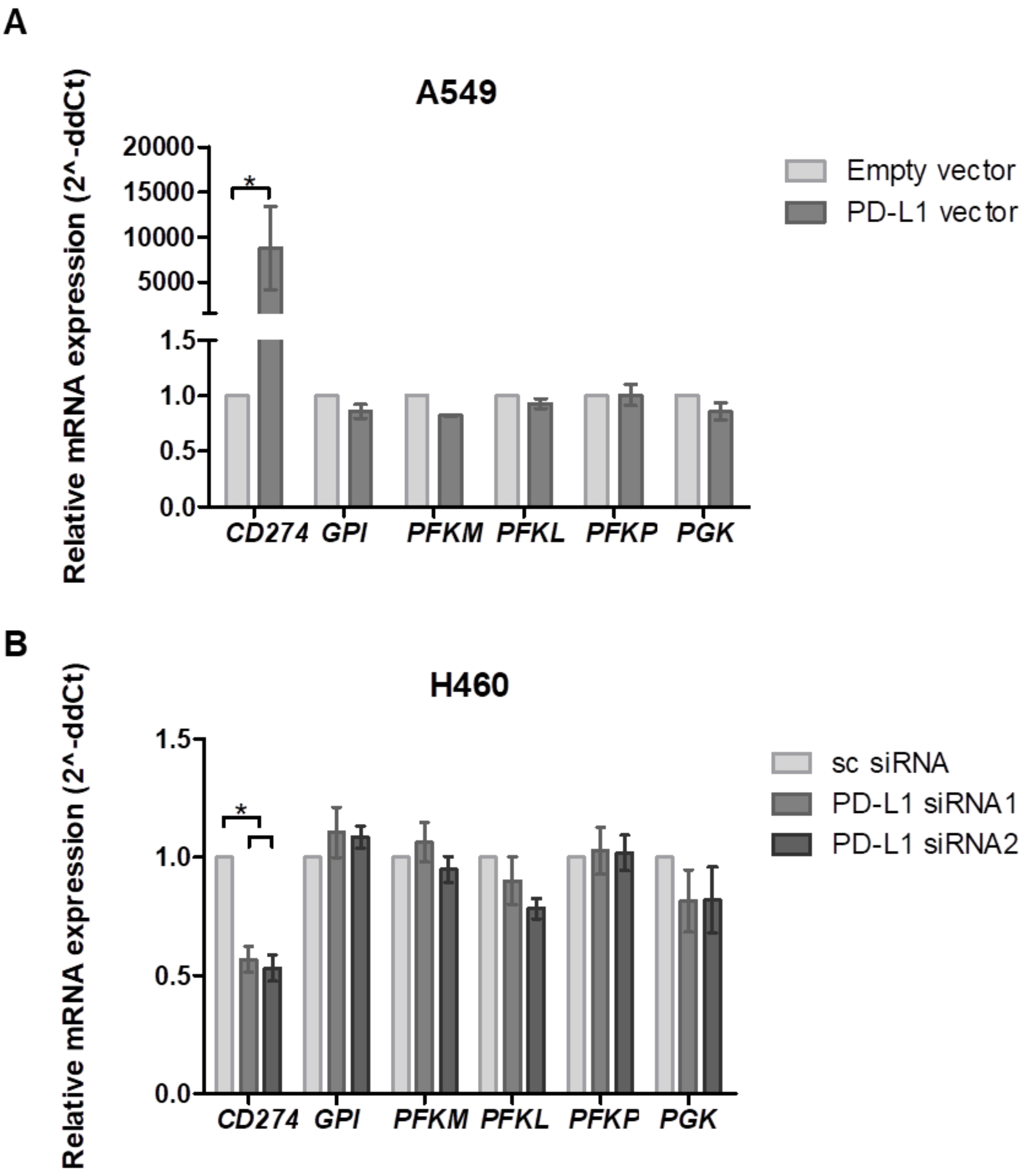

Supplementary Figure S3. PD-L1 overexpression or knockdown does not affect the mRNA level of glycolysis-related genes, other than *HK2*. (A) PD-L1^{low} A549 cells were transfected with empty or PD-L1-expressing vector. Twenty-four hours after transfection, cells were analyzed mRNA level of glycolysis-related genes. **(B)** H460 cells were transfected with scrambled control (sc) siRNA, PD-L1 siRNA1, or PD-L1 siRNA2. Forty-eight hours after transfection, cells were analyzed mRNA level of glycolysis-related genes. Data represent the means ± SEMs of at least three independent experiments. **P*<0.05

Supplementary Figure S4

A. A549 with PD-L1 overexpression (compared to A549 with empty vector)

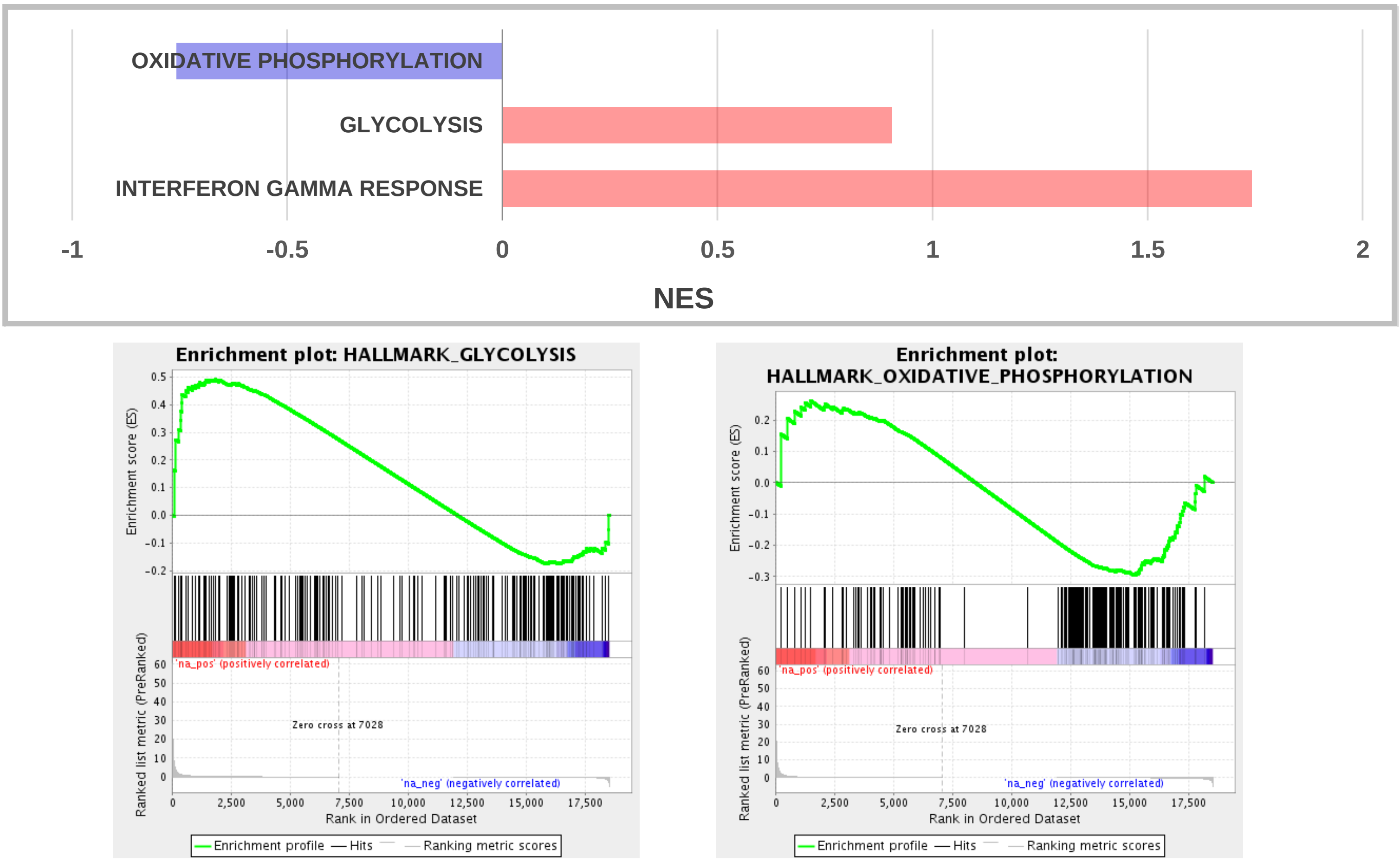

B. H460 with PD-L1 knockdown (compared to H460 with sc siRNA)

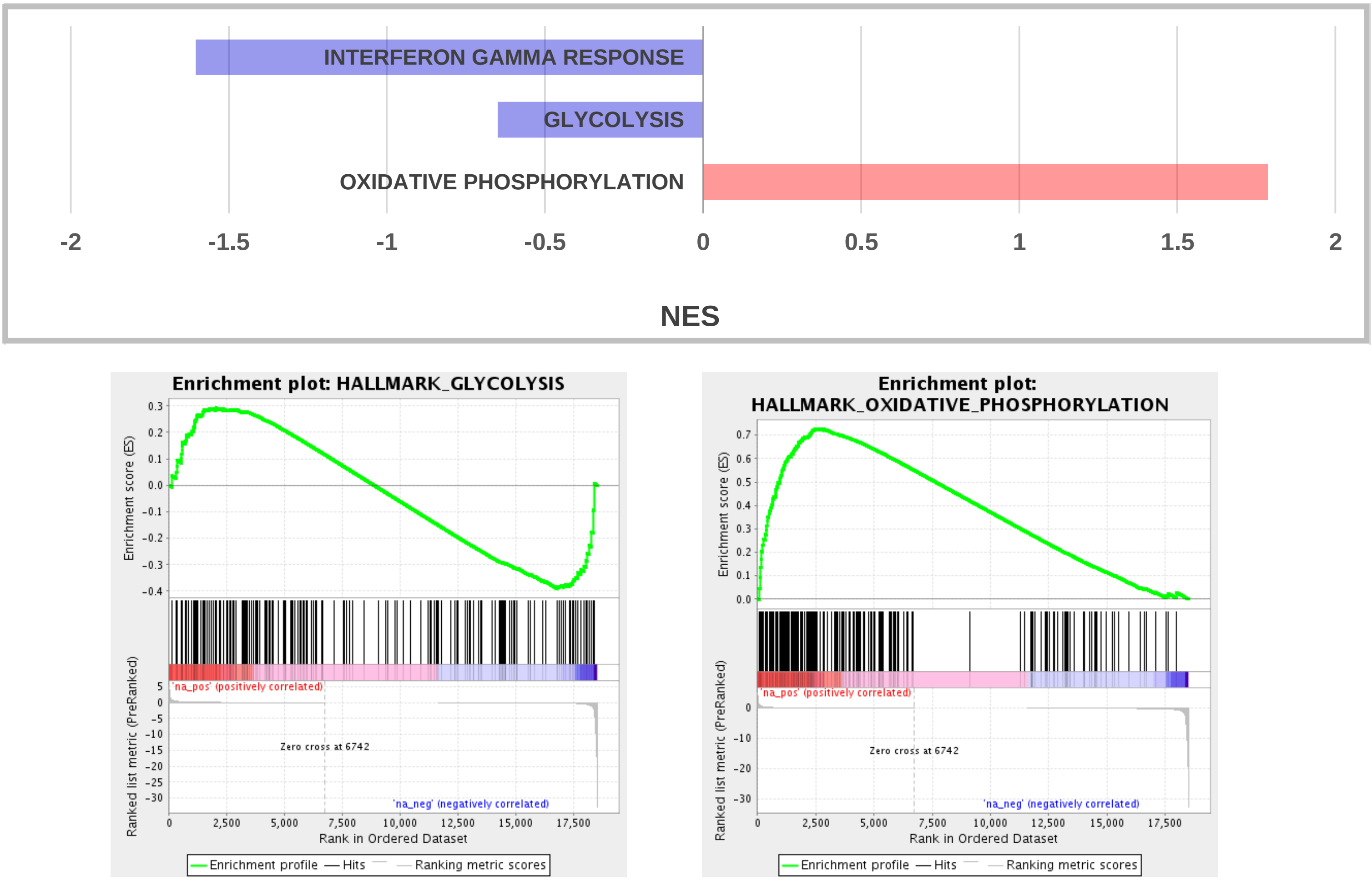

Supplementary Figure S4 (*continued)*

Supplementary Figure S4 PD-L1 expression is positively correlated with glycolysis signature in NSCLC cells. (A) PD-L1^{low} A549 cells were transfected with empty vector or PD-L1-expressing vector. Twenty-four hours after transfection, cells were submitted for transcriptome analyses using RNA seq followed by gene set enrichment analyses (GSEAs). Genes related to glycolysis and the IFN-γ response are enriched but genes related to oxidative phosphorylation are downregulated in A549 cells transfected with PD-L1-expression vector compared to A549 cells transfected with empty vector. **(B)** H460 cells were transfected with scrambled control (sc) siRNA or PD-L1 siRNA1. Forty-eight hours after transfection, cells were submitted for transcriptome analyses using RNA seq followed by GSEA. Genes related to glycolysis and the IFN-γ response were downregulated and genes related to oxidative phosphorylation were enriched in H460 cells transfected with PD-L1 siRNA compared to cells transfected with sc siRNA. Abbreviation: NES, normalized enrichment score.

Supplementary Figure S5

A PET indices according to histology

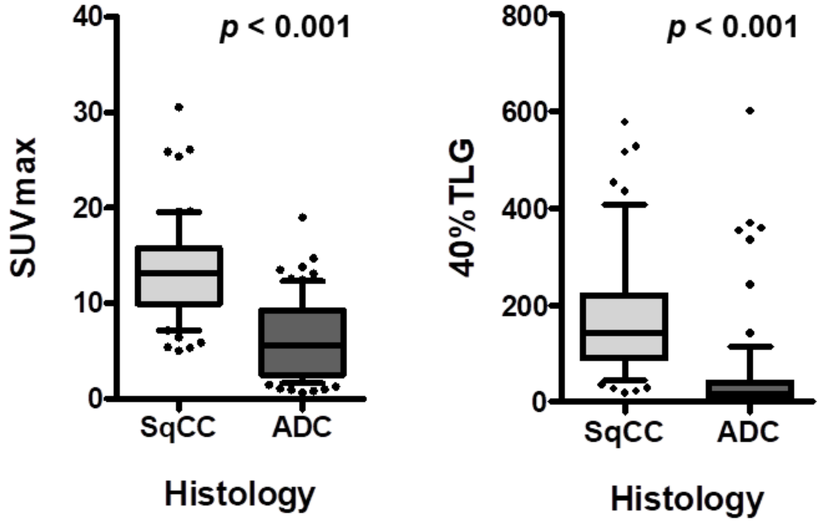

B Basal expression level of glycolysis-related molecules according to histology

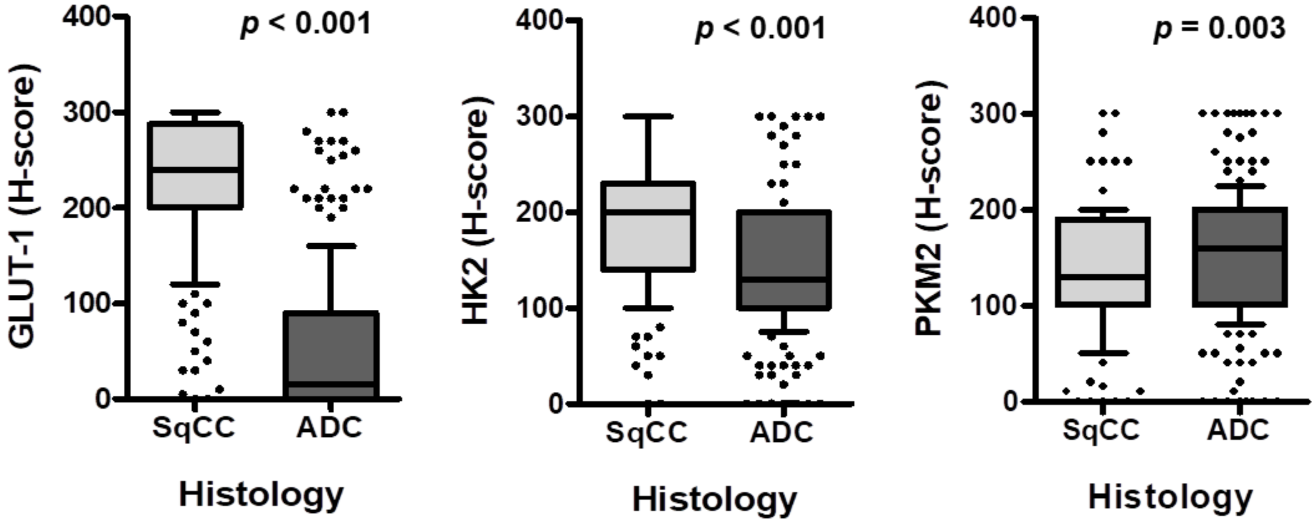

Supplementary Figure S5. Glycolysis-related parameters analyzed by PET scanning and immunohistochemistry in NSCLC patients. (A) PET indices related to glycolysis were compared between patients with pSqCC and pADC. Statistically significant differences were analyzed using Mann Whitney U-tests. **(B)** GLUT-1, HK2 and PKM2 expression levels (H-score) were assessed by immunohistochemistry and compared between patients with pSqCC and pADC. Statistically significant differences were analyzed using Mann Whitney U-tests. Abbreviations: SUVmax, maximal standardized uptake value; TLG, Total lesion glycolysis.

Supplementary Figure S6

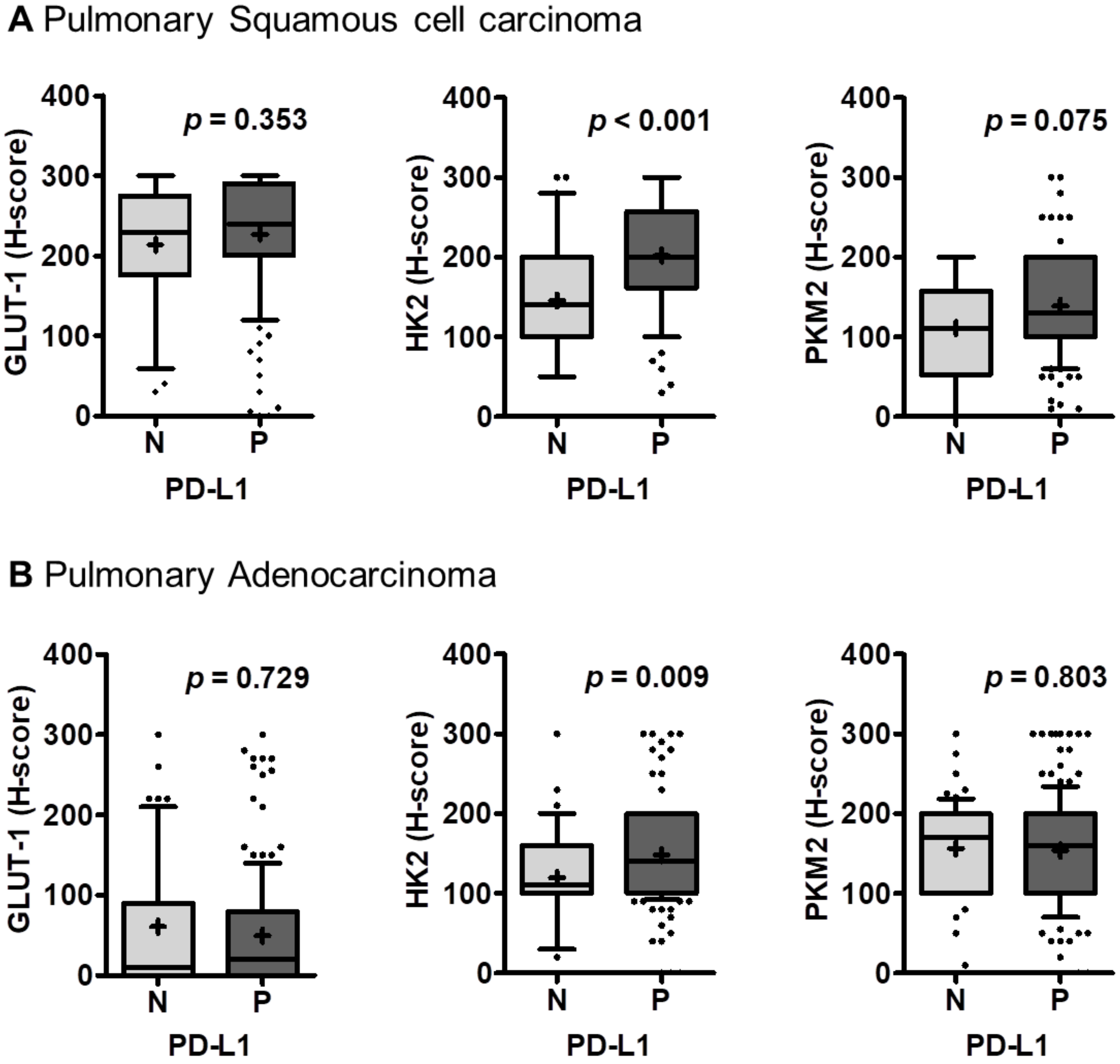

Supplementary Figure S6. Basal expression of glycolysis-related molecules according to PD-L1 expression in pSqCC and pADC patients. Immunohistochemistry analyses for GLUT-1, HK2, and PKM2 were performed in patients with NSCLC (n = 393). The expression (H-score) of these molecules was compared between PD-L1-negative and PD-L1-positive cases in patients with **(A)** pSqCC and **(B)** pADC. Statistically significant differences were analyzed using Mann Whitney U-tests. The whiskers are drawn from the 10th percentile to the 90th percentile. The midline of the box is the median and "+" denotes the mean. Points below and above the whiskers are individual points. Abbreviations: P, positive; N, negative.

Supplementary Figure S7

A. mRNA levels of immune related molecules according to *CD274/SLC2A1* status

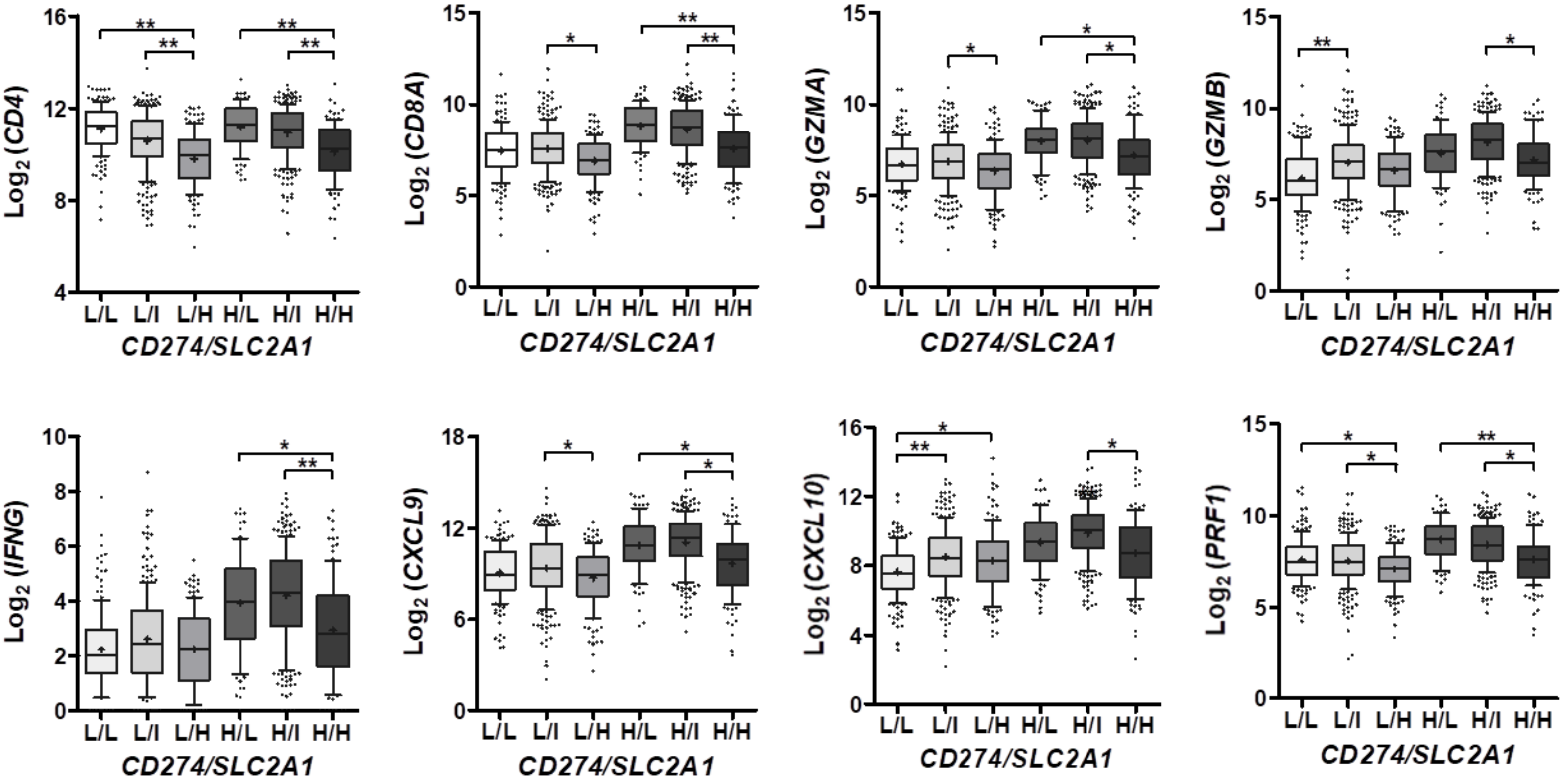

B. mRNA levels of immune related molecules according to *CD274/PKM* status

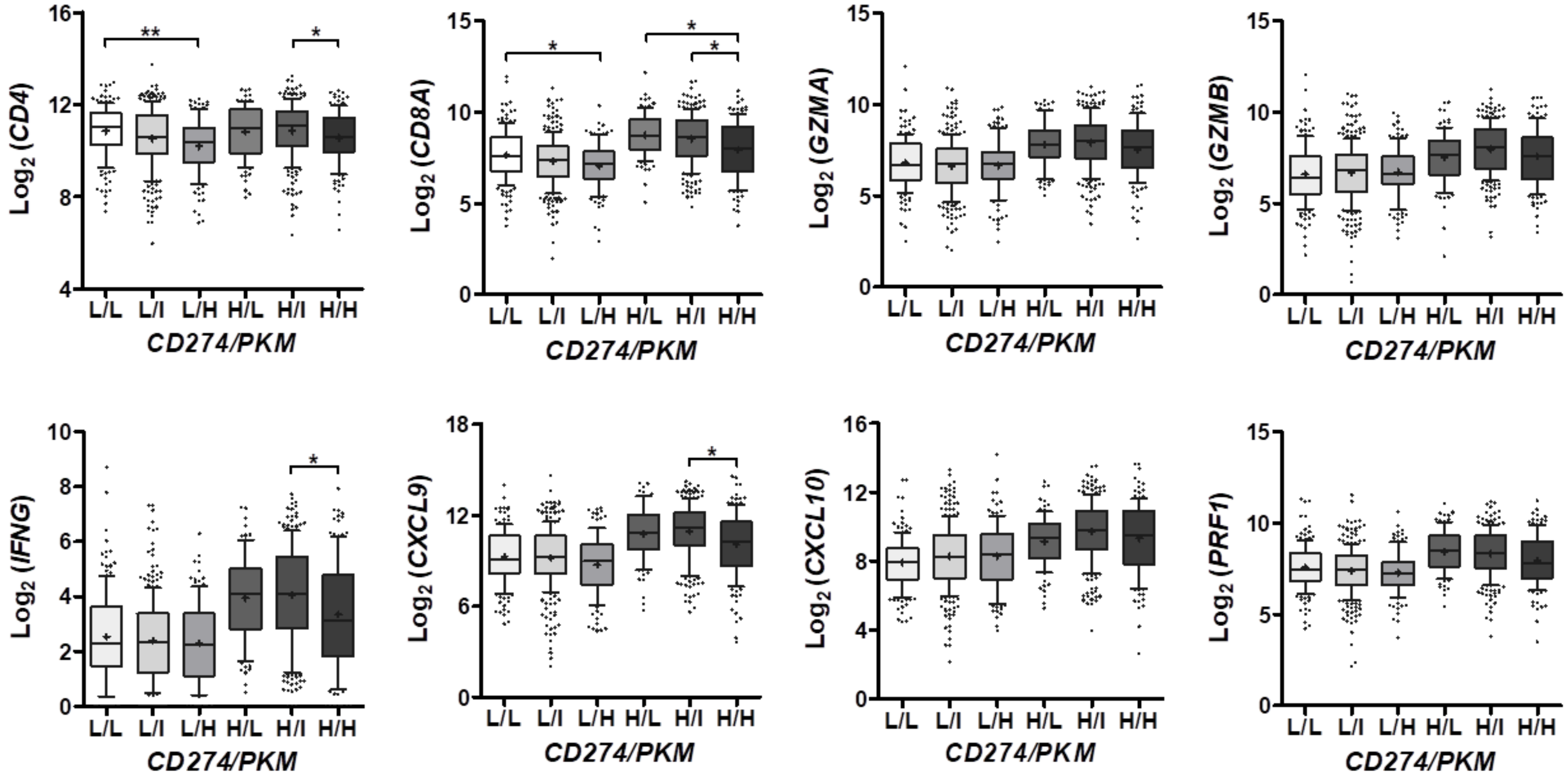

Supplementary Figure S7 continued

Supplementary Figure S7. Expression levels of T-effector immune response-related genes according to *CD274* (PD-L1*) and SLC2A1* (GLUT-1*)* or *PKM* expression status in NSCLC from TCGA data. The expression levels of T-cell effector molecules, including *CD4*, *CD8A*, *GZMA*, *GZMB*, *IFNG*, *CXCL9*, *CXCL10* and *PRF1*, were comparatively analyzed according to **(A)** the *CD274* (PD-L1) and *SLC2A1 (*GLUT-1*)*, or **(B)** the *CD274* (PD-L1) and *PKM* expression status in NSCLCs from TCGA dataset (n = 1015). Cases were dichotomized into PD-$L1^{low}$ and PD-$L1^{high}$ groups based on the median values, and then trichotomized into *SLC2A1* or *PKM* low (<25th percentile), intermediate (25-75th percentile), and high (>75th percentile) groups. Statistically significant differences were analyzed using Kruskal-Wallis tests. *$p < 0.05$; **$p < 0.001$. Abbreviations: L, low; I, intermediate; H, high.

Supplementary Figure S8

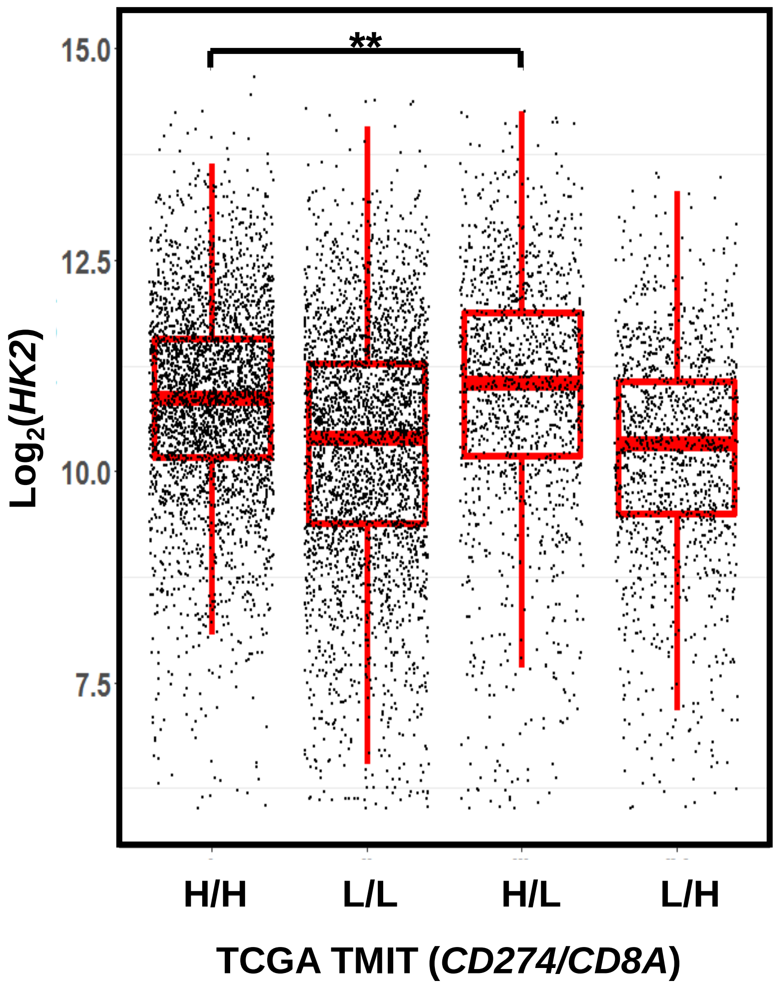

Supplementary Figure S8. HK2 mRNA is higher in TIMT III (CD274^{high}/HK2^{low}) than in TIMT I (CD274^{high}/HK2^{high}) NSCLC from TCGA data. *HK2* transcript levels were compared according to TMIT based on *CD274* (PD-L1)/*CD8A* expression status in NSCLC from TCGA dataset. Statistical differences were analyzed using Kruskal-Wallis tests. $**p < 0.001$. Abbreviations: L, low; H, high.

Supplementary Figure S9

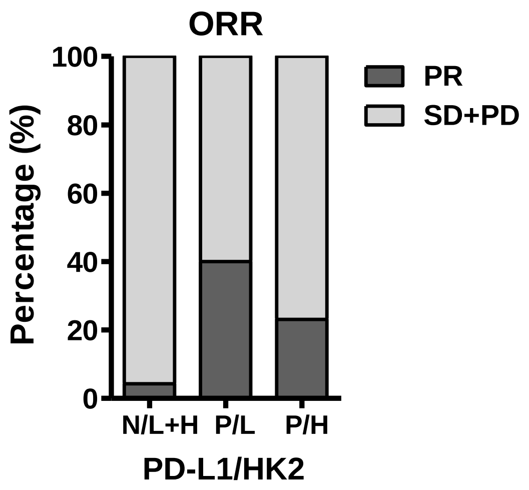

Supplementary Figure S9. High HK2 expression is related to a lower response rate to PD-1 blockade in patients with NSCLC. The response rates to PD-1 blockade in patients with NSCLC according to PD-L1/HK2 expression status. Statistical significance was calculated using Fisher's exact tests. Abbreviations: N, negative; P, positive; L, low; H, high; ORR, overall response rate; PR, partial response; SD, stable disease; PD, progressive disease.

Supplementary Figure S10

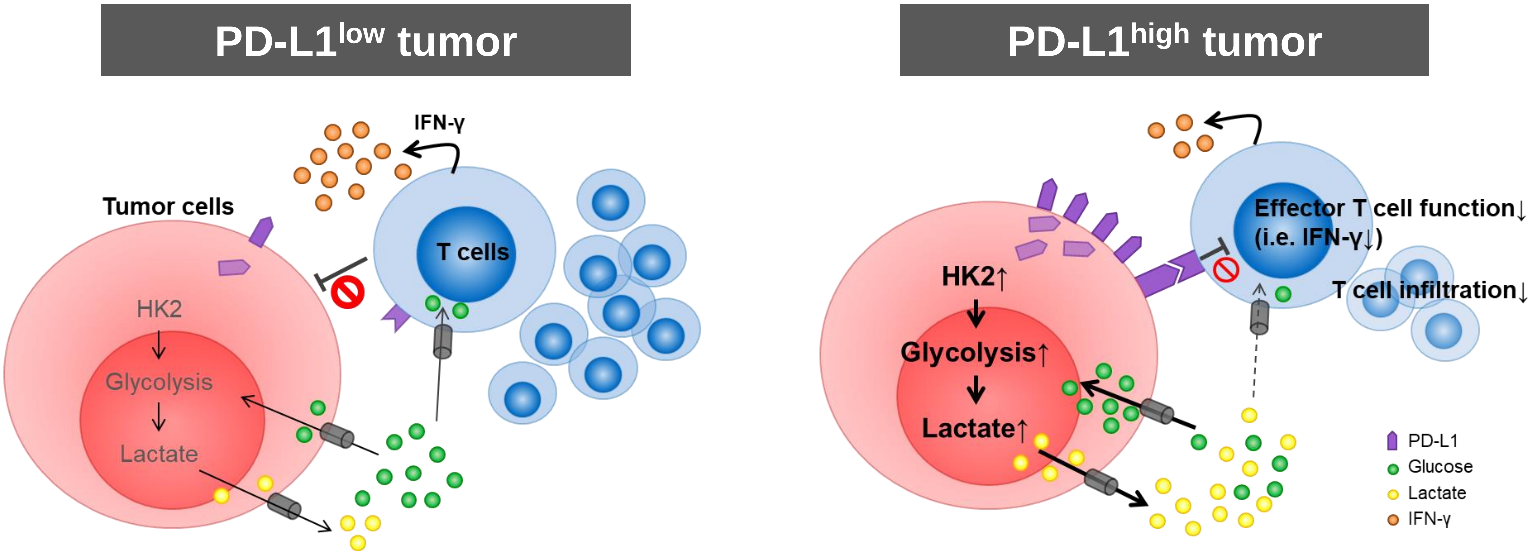

Supplementary Figure S10. A model figure of this study. PD-L1^{high} tumor cells enhance glycolysis by upregulating HK2 expression. Enhanced glucose consumption and lactate production by tumor cells render metabolically harmful environment for surrounding immune cells. In this study, PD-L1^{high} tumors have a relatively low infiltration of CD8^{+} T cells in human lung cancer tissues and relatively low effector T cell functions (i.e. lower IFN-γ secretion), compared to PD-L1^{low} tumors. Therefore, PD-L1^{high} tumor cells outcompete surrounding tumor infiltrating lymphocytes through 1) giving a direct inhibitory signal by PD-L1 and PD-1 ligation and 2) making metabolic harmful environment by enhancing their glycolysis.
